# Supplementary material for: The human gut Firmicute Roseburia intestinalis is a primary degrader of dietary β-mannans
Source: Nat Commun. 2019 Feb 22;10:905. doi: 10.1038/s41467-019-08812-y (PMC6385246; doi:10.1038/s41467-019-08812-y)
Supplement: Supplementary file 1 — Supplementary Information [file 41467_2019_8812_MOESM1_ESM.pdf]

Supplementary Information for

The Human Gut Firmicute *Roseburia intestinalis* is a Primary Degrader of Dietary β-Mannans

La Rosa et al.

Supplementary Table 1. Upregulation of MULL and MULS genes was not detected when *R. intestinalis* L1-82 grew on galactose.

Locus tag	Log_2-fold[a]
ROSINTL182_07683	-0.11
ROSINTL182_07684	-0.03
ROSINTL182_07685	0.03
ROSINTL182_05469	0.43
ROSINTL182_05470	-0.21
ROSINTL182_05471	-0.24
ROSINTL182_05473	-0.27
ROSINTL182_05474	-0.38
ROSINTL182_05475	-0.78
ROSINTL182_05476	-1.01
ROSINTL182_05477	-0.49
ROSINTL182_05478	-1.38
ROSINTL182_05479	-0.63
ROSINTL182_05480	-0.20
ROSINTL182_05481	-0.01
ROSINTL182_05482	-0.11
ROSINTL182_05483	-0.95

[a]Log_2-fold changes of the putative β-mannan utilization genes (as shown in Fig. 1d) expressed by *R. intestinalis* cells grown on galactose relative to glucose.

Supplementary Table 2. Sequence similarity between *R. intestinalis* L1-82's proteins involved in β-mannan utilization and other *Roseburia* strains/species.

		R. intestinalis M50/1	*R. intestinalis* XB6B4	*R. faecis* M72/1	*R. hominis* A2-183	*R. inulinovorans* DSM16841	*R. cecicola* GM
**Locus Tag**	**Function**	**Amino Acids Similarity (%) to *R. intestinalis* L1-82**					
**05470/69**	GH1	99	99	83	92	90	0
**05471**	CEX	99	99	75	76	0	0
**05473**	CE2	99	98	76	75	0	0
**05474**	GH130_2	99	100	85	95	0	0
**05475**	GH130_1	100	100	95	98	0	0
**05476**	Mep	99	99	85	89	0	0
**05477**	MPP2	99	99	89	90	57	0
**05478**	MPP1	100	100	93	89	50	0
**05479**	MnBP	99	100	81	75	0	0
**05480**	TR	99	98	76	79	48	0
**05481**	GH36	99	99	78	82	63	0
**05482**	Pgm	99	100	92	94	94	0
**05483**	GH113	99	99	76	70	0	0
**07683**	GH26	99	99	64	0	0	0
**07684**	GH3B	99	99	62	76	76	0
**07685**	GH3A	99	99	62	76	76	0

Supplementary Table 3. Homologs of *Ri*GH26 (catalytic domain only)[a,b].

Strain	Accession Number	Coverage	Identity	Length[c]	CBM27	CBM23
R. intestinalis XB6B4	CBL14297.1	100%	99%	1308	Y	Y
R. intestinalis M50/1	CBL07467.1	100%	99%	1314	Y	Y
R. faecis	WP_055067491.1	80%	47%	1374	Y	Y
Roseburia sp. CAG:18_43_25	OLA58885.1	77%	48%	1519	Y	Y
Coprococcus eutactus CAG:665	CCZ93652.1	85%	48%	1555	Y	Y
Coprococcus sp. CAG:131	CDB80322.1	85%	47%	1544	Y	Y
Clostridium butyricum	WP_058146428.1	72%	40%	1444	Y	Y
Clostridium disporicum	CUO64427.1	72%	38%	1333	Y	Y
Clostridium sp. Marseille-P2414	WP_066889682.1	73%	37%	1147	Y	Y
Clostridium beijerinckii	WP_026885856.1	69%	39%	1398	Y	Y
Clostridium saudiense	WP_052330667.1	80%	35%	1148	Y	Y
Clostridium puniceum	WP_077847551.1	64%	38%	1016	Y	Y
Propionispora vibrioides	WP_091746906.1	68%	36%	889	Y	Y
Clostridium sp. DSM 8431	WP_090011638.1	66%	36%	1692	Y	Y
Selenomonas bovis	WP_052177425.1	64%	35%	891	Y	Y
Anaerocolumna aminovalerica	SFO28893.1	64%	35%	928	Y	Y

[a]Homologs were identified by BLAST searches against the NCBI non-redundant protein sequence database. [b]Y indicates the presence of a specific CBM in the protein sequence. [C]Amino Acids

Supplementary Table 4. Homologs of *Ri*CBM27[a].

Strain	Phylum	Accession Number	E- value	Coverage	Identity
R. intestinalis XB6B4	Firmicutes	CBL14297.1	2.00E-101	100%	99%
R. intestinalis M50/1	Firmicutes	CBL07467.1	2.00E-100	100%	99%
Coprococcus eutactus CAG:665	Firmicutes	CCZ93652.1	2.00E-23	100%	41%
Coprococcus sp. CAG:131	Firmicutes	CDB80322.1	8.00E-23	100%	40%
R. faecis	Firmicutes	WP_055067491.1	2.00E-22	90%	41%
Roseburia sp. CAG:18_43_25	Firmicutes	OLA58885.1	3.00E-22	90%	41%
Clostridium disporicum	Firmicutes	CUO64427.1	1.00E-18	98%	34%
Clostridium butyricum	Firmicutes	WP_058146428.1	7.00E-18	93%	39%
Clostridium sp. Marseille-P2414	Firmicutes	WP_066889682.1	2.00E-17	98%	33%
Clostridium puniceum	Firmicutes	WP_077847551.1	4.00E-17	91%	34%
Uncultured *Clostridium sp.*	Firmicutes	SCJ87681.1	3.00E-15	91%	32%
Clostridium saudiense	Firmicutes	WP_052330667.1	4.00E-15	91%	32%
Clostridium beijerinckii	Firmicutes	WP_026885856.1	3.00E-11	98%	33%
Anaerocolumna aminovalerica	Firmicutes	SFO28893.1	6.00E-11	96%	32%
Selenomonas bovis	Firmicutes	WP_052177425.1	9.00E-11	90%	31%
Clostridium sp. DSM 8431	Firmicutes	WP_090011638.1	2.00E-10	98%	30%
Propionispora vibrioides	Firmicutes	WP_091746906.1	8.00E-10	91%	32%
Pelosinus fermentans JBW45	Firmicutes	AJQ29746.1	3.00E-09	92%	31%

[a]Homologs were identified by BLAST searches against the NCBI non-redundant protein sequence database.

Supplementary Table 5. Homologs of *Ri*CBM23[a].

Strain	Phylum	Accession Number	E- value	Coverage	Identity
R. intestinalis XB6B4	Firmicutes	CBL14297.1	1.00E-123	100%	99%
R. intestinalis M50/1	Firmicutes	CBL07467.1	1.00E-123	100%	99%
Coprococcus eutactus CAG:665	Firmicutes	CCZ93652.1	5.00E-58	93%	58%
Coprococcus sp. CAG:131	Firmicutes	CDB80322.1	2.00E-57	93%	58%
R. faecis	Firmicutes	WP_055067491.1	3.00E-44	88%	49%
Roseburia sp. CAG:18_43_25	Firmicutes	OLA58885.1	4.00E-43	88%	49%
Clostridiales bacterium CHKCI001	Firmicutes	CVI73099.1	4.00E-41	93%	44%
Clostridium beijerinckii	Firmicutes	WP_026885856.1	9.00E-40	89%	47%
Clostridium butyricum	Firmicutes	WP_058146428.1	5.00E-36	94%	42%
Propionispora vibrioides	Firmicutes	WP_091746906.1	2.00E-34	99%	43%
Clostridium puniceum	Firmicutes	WP_077847551.1	2.00E-35	89%	41%
Selenomonas bovis	Firmicutes	WP_052177425.1	2.00E-33	89%	42%
Clostridium sp. DSM 8431	Firmicutes	WP_090011638.1	4.00E-33	93%	40%
Clostridium disporicum	Firmicutes	WP_055276275.1	2.00E-30	94%	41%
Clostridium sp. Marseille-P2414	Firmicutes	WP_066889682.1	3.00E-30	91%	40%
Clostridium saudiense	Firmicutes	WP_052330667.1	5.00E-30	96%	41%
Clostridium populeti	Firmicutes	WP_092560634.1	2.00E-21	92%	33%
Lachnospiraceae bacterium ND2006	Firmicutes	WP_051666040.1	2.00E-21	100%	34%
Cohnella sp. OV330	Firmicutes	WP_090116078.1	1.00E-18	89%	36%
Anaerocolumna aminovalerica	Firmicutes	SFO28893.1	3.00E-17	99%	32%

[a]Homologs were identified by BLAST searches against the NCBI non-redundant protein sequence database.

Supplementary Table 6. Carbohydrates used in this study, listed with supplier and product number.

Substrate	Source	Catalogue
Glucose	Sigma	(G8270)
Galactose	Sigma	(G0750)
Mannose	Sigma	(M4625)
Mannobiose	Megazyme	(O-MBI)
Mannotriose	Megazyme	(O-MTR)
Mannotetraose	Megazyme	(O-MTE)
Mannopentaose	Megazyme	(O-MPE)
Mannohexaose	Megazyme	(O-MHE)
Cellobiose	Sigma	(C7252)
Cellotriose	Megazyme	(O-CTR)
Cellotetraose	Megazyme	(O-CTE)
Cellopentaose	Megazyme	(O-CPE)
Cellohexaose	Megazyme	(O-CHE)
Mannose-1-phosphate	Sigma	(M1755)
Mannose-6-phosphate	Sigma	(M6876)
Glucose-1-phosphate	Sigma	(G7000)
Glucose-6-phosphate	Sigma	(G7879)
Fructose-6-phosphate	Sigma	(F3627)
Glucosylmannose plus Mannobiose	Megazyme	(O-GMMBI)
6^1-α-D-Galactosylmannobiose plus Mannotriose	Megazyme	(OGMM-3)
6^1-α-D-Galactosylmannotriose	Megazyme	(O-GM3)
6^3,6^4-α-D-Galactosylmannopentaose	Megazyme	(O-GGM5)
Galactomannan (guar gum)	Sigma	(G4129)
Galactomannan (carob)	Megazyme	(P-GALML)
Glucomannan (konjac)	Megazyme	(P-GLCML)
Acetylated galactoglucomannan (Spruce)	This study[#]	
Birch xylan	This study[#]	
Lichenan (icelandic moss)	Megazyme	(P-LICHN)
β-glucan (barley)	Megazyme	(P-BGBL)
Curdlan	Megazyme	(P-CURDL)
Wheat arabinoxylan	Megazyme	(P-WAXYL)

[#]Produced at the Bioprocess Technology and Biorefining Lab, Faculty of Chemistry, Biotechnology and Food Science, Norwegian University of Life Sciences, according to procedure described in[1].

Supplementary Table 7. Primers, vector for overexpression in *E. coli* and protein purification techniques used in this study[a].

Gene	Primer (5’ - 3’)	Vector	Purif. Tech.[b]
ROSINTL182_07683<br>(CBM27/GH26/CBM23)	**F:**TTAAGAAGGAGATATACTATGGGGGGATACGAGTATGTCTATGC<br>**R:**AATGGTGGTGATGATGGTGCGCTCCACCTTCTGTCACATTTAAC	pNIC-CH	IMAC/SEC
ROSINTL182_07683<br>(CBM27)	**F:**TTAAGAAGGAGATATACTATGGGGGGATACGAGTATGTCTATGC<br>**R:**AATGGTGGTGATGATGGTGCGCGCCCGCTGCATTATACTGG	pNIC-CH	IMAC/SEC
ROSINTL182_07683<br>(CBM23)	**F:**TTAAGAAGGAGATATACTATGTCTGTTTTTGCGAATGGAACAAAC<br>**R:**AATGGTGGTGATGATGGTGCGCTCCGGACACGAGTGACTGATC	pNIC-CH	IMAC/SEC
ROSINTL182_07684	**F:**TTAAGAAGGAGATATACTATGGGTAACAGTGGAATCGGTGTTC<br>**R:**AATGGTGGTGATGATGGTGCGCCTCTAACTCAATCCAGTCCAACTTC	pNIC-CH	IMAC/SEC
ROSINTL182_07685	**F:**TTAAGAAGGAGATATACTATGGAAAAATGGCAAAGATCACTTTATC<br>**R:**AATGGTGGTGATGATGGTGCGCGTCGATTTTCAGTATTAATCCTAAAATAC	pNIC-CH	IMAC/SEC
ROSINTL182_05469<br>(GH1_D2)	**F:**TTAAGAAGGAGATATACTATGATACTTAGTATGAATCAGGCAAATAAAG<br>**R:**AATGGTGGTGATGATGGTGCGCTTTTACAGTAGAAACGATCAGACTCATC	pNIC-CH	IMAC/SEC
ROSINTL182_05470<br>(GH1_D1)	**F:** TTAAGAAGGAGATATACTATGTTAGACCCGGTATGTACGCAC<br>**R:** AATGGTGGTGATGATGGTGCGCTCCTCCATTCGTTTCTATTACTTTCTG	pNIC-CH	IMAC/SEC
ROSINTL182_05471	**F:**TTAAGAAGGAGATATACTATGGAATATCAAATTAAATACGAAAACGGC<br>**R:**AATGGTGGTGATGATGGTGCGCTTTTTCAGAGGAACCAATGACAGAC	pNIC-CH	IMAC/SEC
ROSINTL182_05473	**F:**TTAAGAAGGAGATATACTATGAAACGTGTGATGGAGTGTCCG<br>**R:**AATGGTGGTGATGATGGTGCGCAGATTCCCAGACTGCATCCC	pNIC-CH	IMAC/SEC
ROSINTL182_05474	**F:**TTAAGAAGGAGATATACTATGAGCAAATATAAAATGATCAGCCAGC<br>**R:**AATGGTGGTGATGATGGTGCGCGCGTCTTCCGATCTCGGTATC	pNIC-CH	IMAC/SEC
ROSINTL182_05475	**F:**TTAAGAAGGAGATATACTATGGAGATTCGACGAATGTTACATGAG<br>**R:**AATGGTGGTGATGATGGTGCGCTTTATCTTCCTTTGCAAGGAACTC	pNIC-CH	IMAC/SEC
ROSINTL182_05476	**F:**TTAAGAAGGAGATATACTATGAGTGAGTTAAAAGAACTTGCAGCAG<br>**R:**AATGGTGGTGATGATGGTGCGCGAATCAGTTTTATGCTCCGATTCTG	pNIC-CH	HIC/<br>SEC
ROSINTL182_05479	**F:** TTTCAGGGCGCCATGGGCTCAAACAACACAGCAGG<br>**R:** GACGGAGCTCGAATTTTACTCAGTTACTTCAATTCCGTTTG	pETM-11	IMAC/SEC
ROSINTL182_05481	**F:**TTAAGAAGGAGATATACTATGGCAATCACATACTTTGAAAAAGAACGC<br>**R:**AATGGTGGTGATGATGGTGCGCTTTTTCCATTTAATCTTATTTCTTTTCCAC	pNIC-CH	HIC/<br>SEC
ROSINTL182_05482	**F:**TTAAGAAGGAGATATACTATGAGCAATTACATGGAAACATACAAACAG<br>**R:**AATGGTGGTGATGATGGTGCGCTTCCTACTGTTATTTCAGCAAGTCTTC	pNIC-CH	HIC/<br>SEC
ROSINTL182_05483	**F:**TAAGAAGGAGATATACTATGGAATATATCTGTGGGGTGACTTTTGC<br>**R:**AATGGTGGTGATGATGGTGCGCTTGACCTCAACATCATTTATTAAAAATATC	pNIC-CH	HIC/<br>SEC

[a]The 5’ extension sequences used for molecular cloning are underlined. [b]Purification techniques employed.

Supplementary Table 8. Primers used for sequencing of the intergenic region between ROSINTL182_05470 - 05469.

Gene	Primer (5’ - 3’)	Application
ROSINTL182_05470/<br>ROSINTL182_05469	**F:**GGGATTACGCCATATATTACC<br>**R:**GTCTCCGTTTGGATGTGC	Sequencing of the intergenic region between *Ri*GH1_D1 and *Ri*GH1_D2

Supplementary Table 9. Primers used for qPCR.

Target	Primer (5’ - 3’)	Supplementary Reference
R. intestinalis L1-82	**F:** GCATGACCTGGTGTGAA<br>**R:** TTGGGCCGTGTCTCA	Larsen, N. et al[2]
B. ovatus ATCC 8384	**F:** GGTGTCGGCTTAAGTGCCAT<br>**R:** CGGAYGTAAGGGCCGTGC	Larsen, N. et al[2]
Universal 16S rRNA	**F:** CCTAYGGGRBGCASCAG<br>**R:** GGACTACNNGGGTATCTAAT	Yu, Y. et al[3]

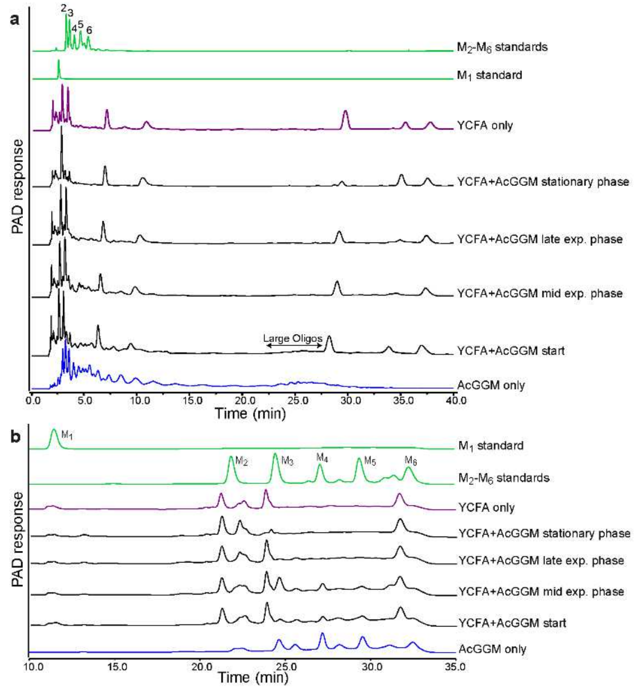

Supplementary Figure 1. *R. intestinalis* L1-82 AcGGM consumption at zero, middle and later time points, determined by HPAEC-PAD. a, Chromatograms showing the oligosaccharides detected in the supernatant of *R. intestinalis* L1-82 while growing on YCFA supplemented with 5 mg ml^{-1} AcGGM. M_2-M_6 standards were 2, mannobiose; 3, mannotriose; 4, mannotetraose; 5, mannopentaose; 6, mannohexaose. **b**, Chromatograms with a gradient adapted to improve the resolution of mono-, di- and shorter oligosaccharides present in the same supernatant as in a. The data are representative of three biological replicates. Abbreviations: mid exp. phase; middle exponential phase; late exp. phase, late exponential phase.

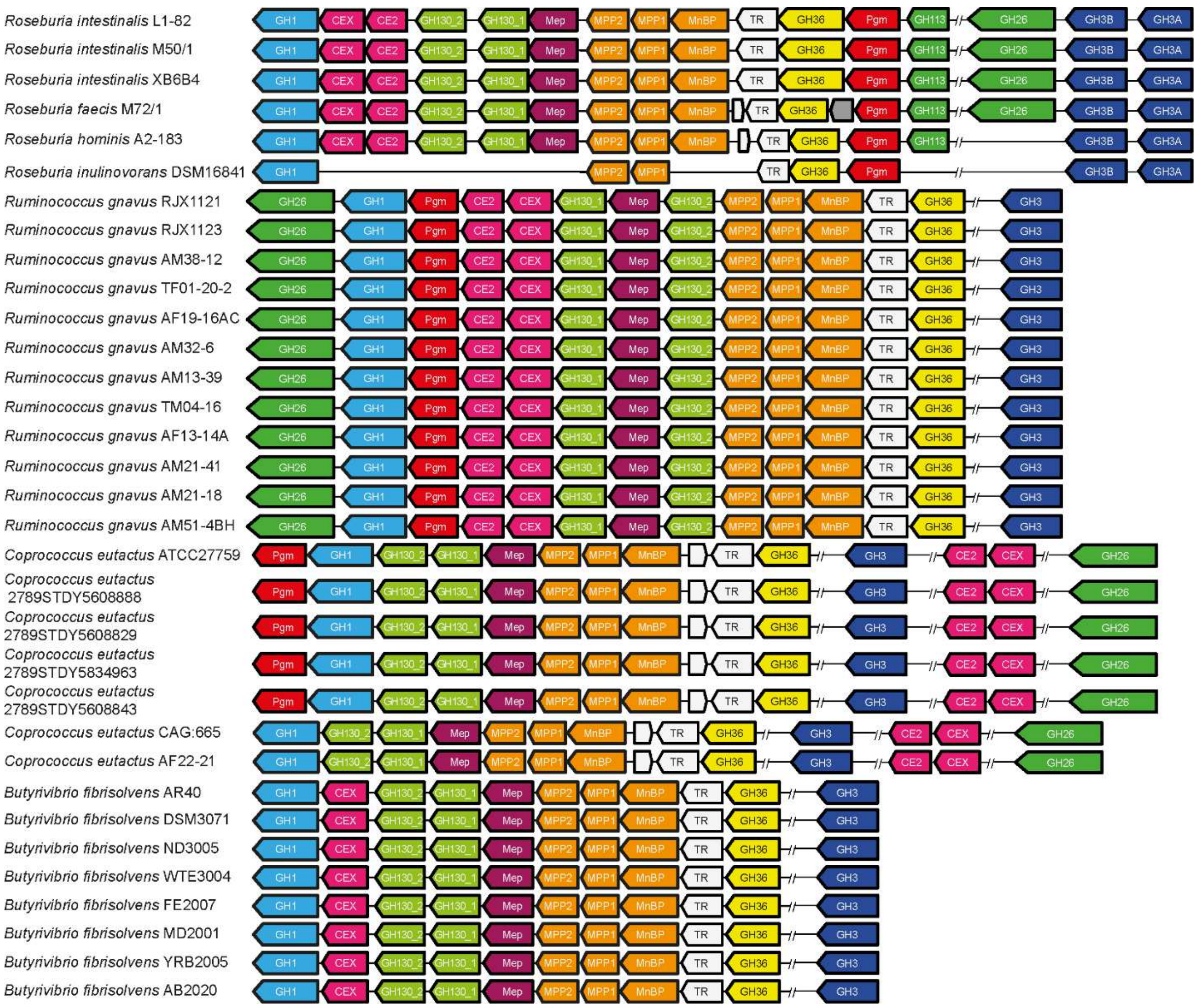

Supplementary Figure 2. Sequence conservation and genomic organization of the β-mannan utilization loci among *Roseburia* species and closely related members of the Clostridium cluster XIVa. Common predicted functions are colored according to Fig. 2d. The figure shows that MULL and MULS are conserved in all the *R. intestinalis* strains, *R. faecis* and *R. hominis* A2-183; however, the latter genome does not include an orthologous GH26 gene. *R. inulinovorans* lacks most of the genes encoding the β-mannan utilization apparatus, suggesting a different carbohydrate utilization specialization for this strain. Among members of the Clostridium cluster XIVa, several *R. gnavus* and *C. eutactus* strains harbor similar MULL and MULS genes, although no GH113 was detected. The mannanase GH26 is lacking from the genomes of the *B. fibrisolvens* strains, suggesting that these bacteria are likely unable to hydrolyze polymeric β-mannans.

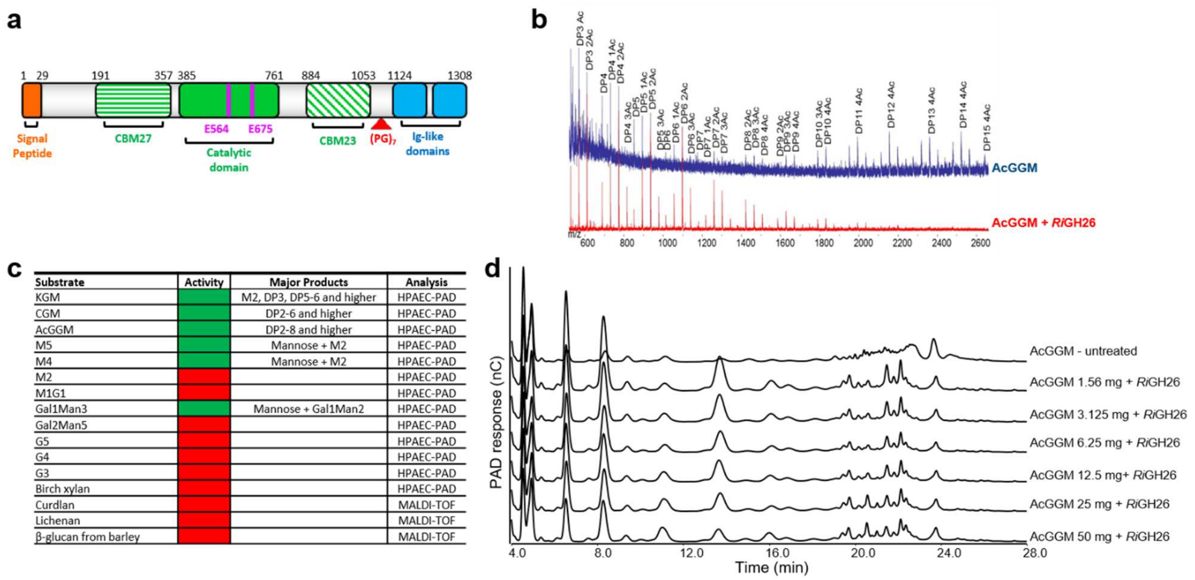

Substrate	Activity	Major Products	Analysis
KGM		M2, DP3, DP5-6 and higher	HPAEC-PAD
CGM		DP2-6 and higher	HPAEC-PAD
AcGGM		DP2-8 and higher	HPAEC-PAD
M5		Mannose + M2	HPAEC-PAD
M4		Mannose + M2	HPAEC-PAD
M2			HPAEC-PAD
M1G1			HPAEC-PAD
Gal1Man3		Mannose + Gal1Man2	HPAEC-PAD
Gal2Man5			HPAEC-PAD
G5			HPAEC-PAD
G4			HPAEC-PAD
G3			HPAEC-PAD
Birch xylan			HPAEC-PAD
Curdlan			MALDI-TOF
Lichenan			MALDI-TOF
β-glucan from barley			MALDI-TOF

Supplementary Figure 3. Schematic of *Ri*GH26 and activity on different glycans. a, Domain organization of *Ri*GH26. The protein contains an N-terminal secretion signal peptide (in orange), as detected by the SignalP 4.1 server (http://www.cbs.dtu.dk/services/SignalP/), which is predicted to be removed by cleavage between Gly29 and Tyr30. The CBMs and catalytic domain are shown in green with the catalytic residues in magenta. The red arrow indicates the position of the Proline-Glycine rich region. The two Ig-like domains are shown in light blue. Based on these predictions, the gene was cloned and the protein expressed without the N-terminal signal, C-terminal PG-rich region and Ig-like sequences. **b**, Activity of *Ri*GH26 on AcGGM. The picture shows the MALDI-TOF spectra of oligosaccharides before (blue) and after *Ri*GH26 treatment (red). **c**, Summary of the activity of *Ri*GH26 on different glycans under standard assay conditions. A green box indicates activity while a red box indicates the absence of detectable products. **d**, Activity of *Ri*GH26 on different concentrations of AcGGM. *Ri*GH26, at 10 nM, was incubated with increasing concentration of AcGGM for 1 h in standard assay conditions. The reaction products were diluted to have a final concentration of approximately 1 mg ml^{-1} and subjected to HPAEC-PAD.

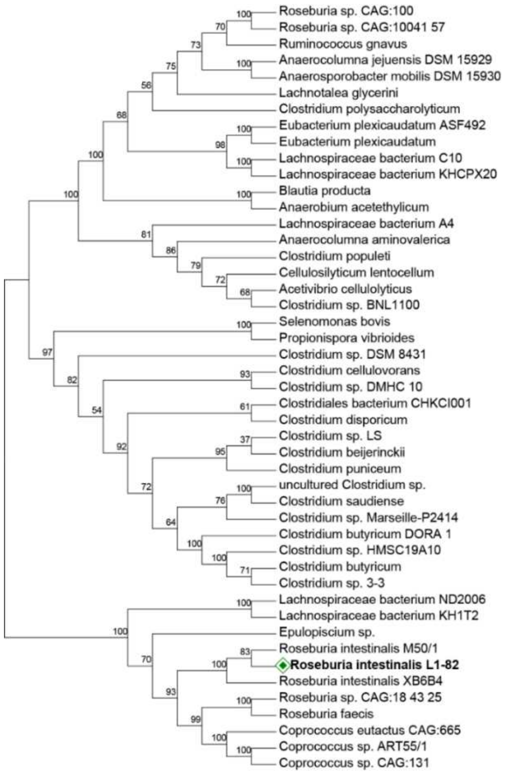

Supplementary Figure 4. Unrooted maximum likelihood tree of *Ri*GH26 and homologs. Searching for *Ri*GH26-like proteins was performed by BLAST against the NCBI non-redundant protein sequence database. Sequences with coverage <86% and identity <40% were eliminated. Multiple sequence alignment was performed using ClustalW. The phylogenetic tree was generated using the maximum likelihood algorithm in Mega 7 (http://megasoftware.net/). Bootstrap values are shown at each node as percentage of 500 replicates. *Ri*GH26 is indicated by a green-colored diamond. All sequences were identified as members of the Firmicutes phylum, with 96% of the sequence belonging to the order Clostridiales. Exceptions were the sequence of *Selenomonas bovis* and *Propionospora vibrioides* that belong to the class Negativicutes. The majority of the sequences were affiliated to the Clostridium Cluster XIVa.

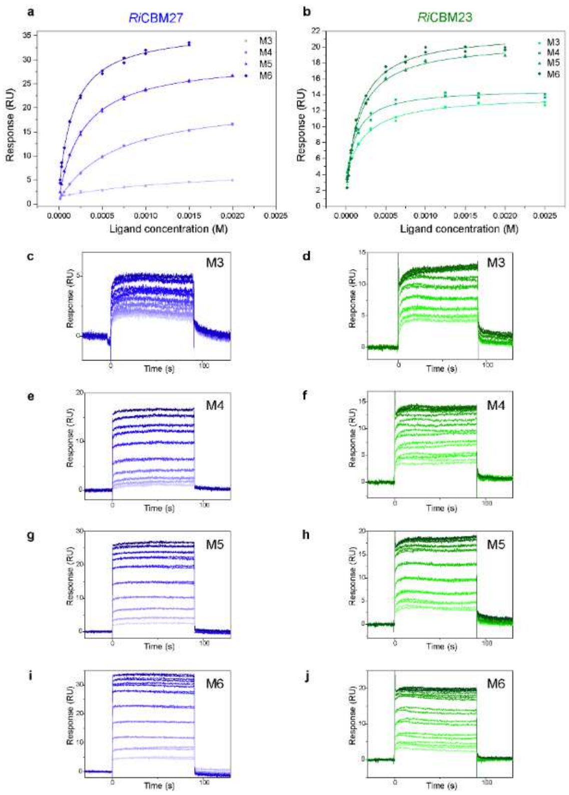

Supplementary Figure 5. Binding of *Ri*CBM27 and *Ri*CBM23 to manno-oligosaccharides using SPR analysis (related to Table 1). Blank and reference cell corrected (**a**-**b**) and raw SPR sensograms (**c**-**j**) showing the binding of *Ri*CBM27 (blue) and *Ri*CBM23 (green) to manno-oligosaccharides (M3-6). In all panels, the results are representative of at least two independent replicates.

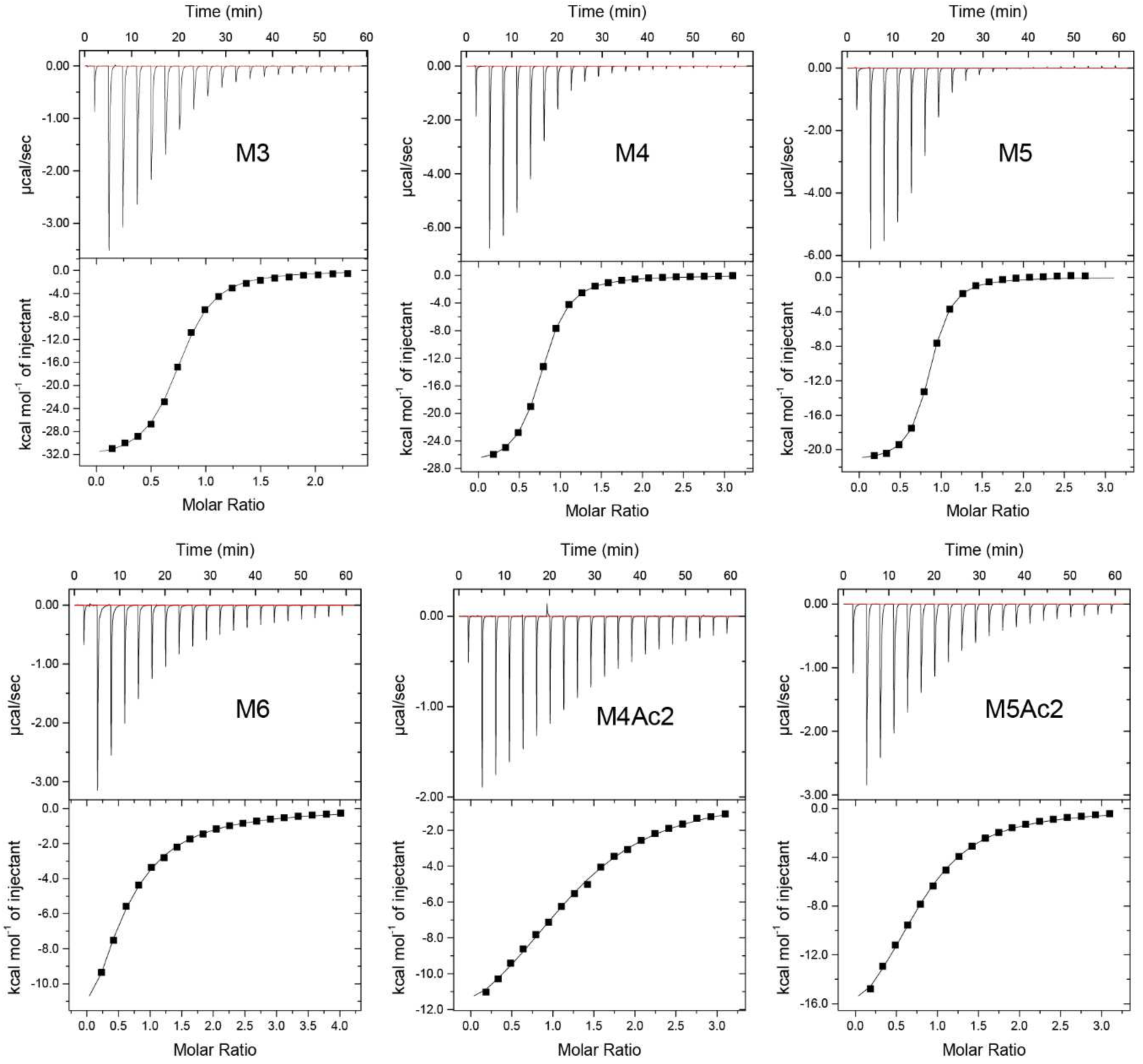

Supplementary Figure 6. ITC analysis of *Ri*MnBP binding to linear and substituted manno-oligosaccharides (related to Table 2). Data show *Ri*MnBP binding to manno-oligosaccharides (M3-6), diacetylated mannotetraose (M4Ac2) and diacetylated mannopentaose (M5Ac2). The top panel in each pair shows the thermograms, whereas the bottom graph depicts the binding isotherms and one set of equivalent binding sites model fits to the data (solid lines).

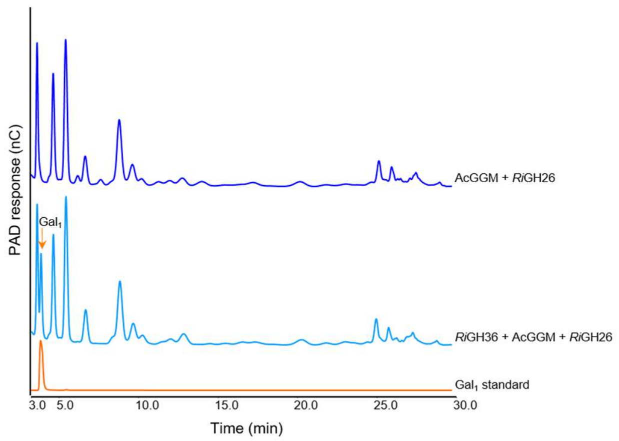

Supplementary Figure 7. Activity of *Ri*GH36 on AcGGM. HPAEC-PAD analysis of *Ri*GH26-treated AcGGM before and after subsequent incubation with *Ri*GH36.

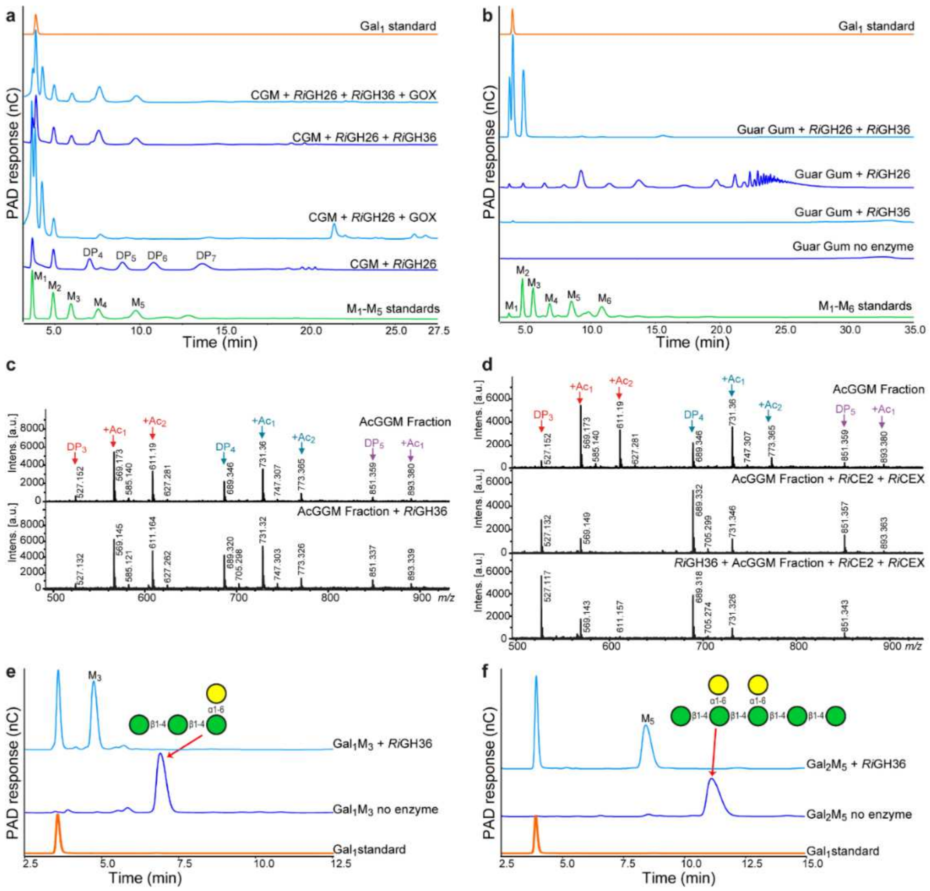

Supplementary Figure 8. Activity of *Ri*GH36 on galactomannan, acetylated manno-oligosaccharides and galactomanno-oligosaccharides. a, HPAEC-PAD chromatograms showing the effect of the treatment with galactose oxidase (GOX from *Dactylium dendroides*, Megazyme) on CGM pre-hydrolyzed with *Ri*GH26 and subsequent incubation with *Ri*GH36. The oligosaccharides (DP4-7) released from CGM by *Ri*GH26 were oxidized by the GOX; indeed, upon GOX treatment, the galactosylated oligosaccharides at denoted DP4-7 disappear and the corresponding oxidized oligosaccharides appear at 20+ minutes. No oxidized product could be observed after GOX-treatment of the *Ri*GH26 and *Ri*GH36-treated CGM sample, demonstrating that *Ri*GH36 completely removes the galactose residues from the substrates. **b**, HPAEC-PAD analysis of the products released from guar gum galactomannan by *Ri*GH36 alone or when the enzyme is combined with the β-mannanase *Ri*GH26. The figure shows that *Ri*GH36 has limited activity on the polymeric substrate while it acts synergistically with *Ri*GH26 to degrade the guar gum galactomannan completely into mannose, galactose and mannobiose. **c**, MALDI-TOF spectra of the hydrolysis of a preparation containing trisaccharides (DP3), tetrasaccharides (DP4) and pentasaccharides (DP5) with different degree of acetylation (Ac), after overnight incubation with *Ri*GH36. **d**, Increased *Ri*GH36 activity can be observed as an increase in DP3 and a corresponding reduction in DP4 and DP5 when the same substrate as in panel c was pretreated with *Ri*CE2 and *Ri*CEX to remove the acetyl substitutions. **e**, HPAEC-PAD analysis of the reaction products generated after *Ri*GH36 treatment of Gal_1M_3 (15 mM). **f**, Products released from Gal_2M_5 (8.7 mM) by *Ri*GH36 action as analyzed by HPAEC-PAD. In all panels, *Ri*GH36 was used at a concentration of 15 nM and incubated with the glycans for 16 h at 37 ºC.

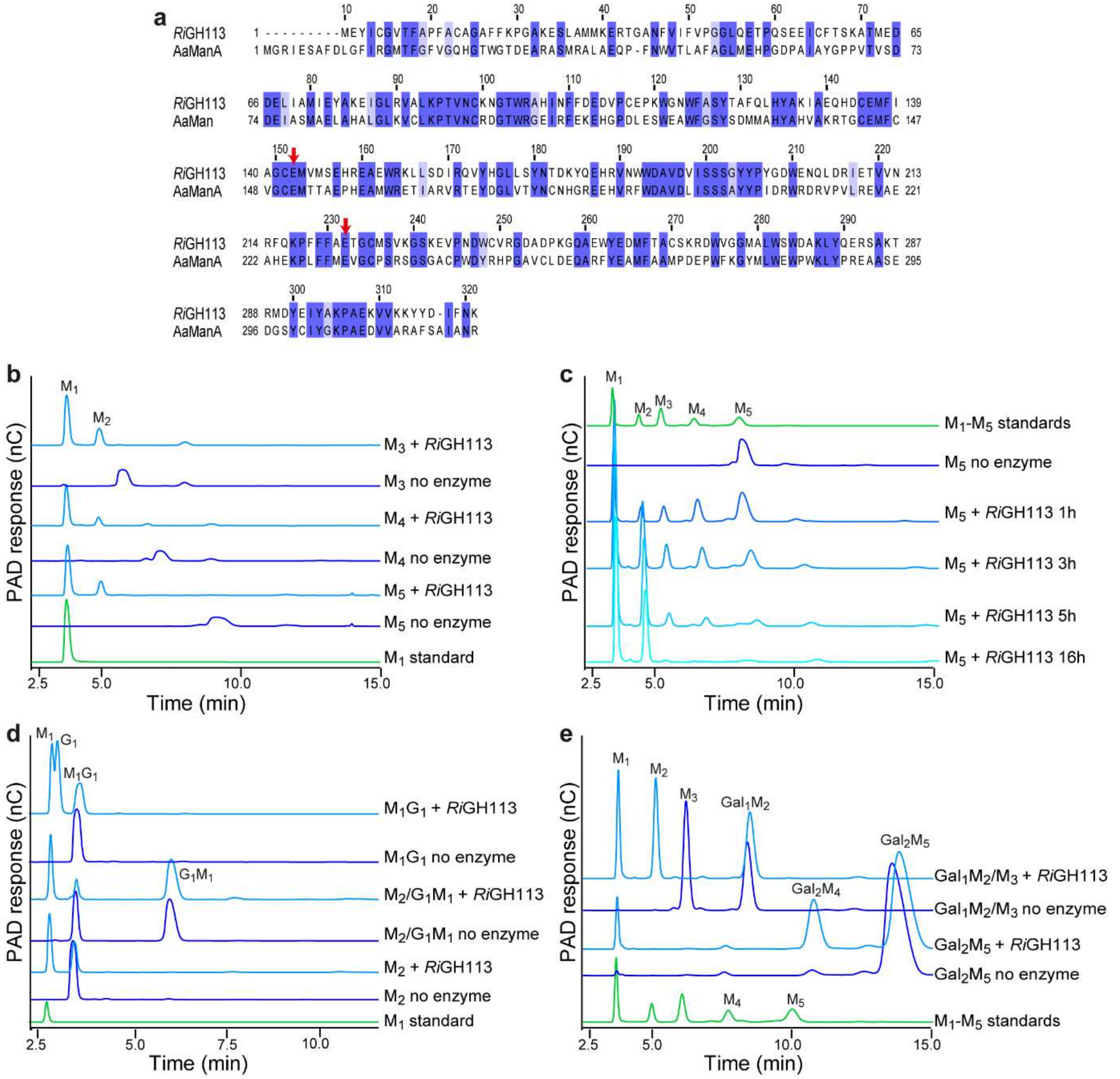

Supplementary Figure 9. Sequence analysis, activity of *Ri*GH113 towards various manno-oligosaccharides and time course activity. a, Amino acid sequence alignment of the GH113 protein of *R. intestinalis* L1-82 (*Ri*GH113) and AaManA from *Alicyclobacillus acidocaldarius*. The protein sequences were aligned with JalView (http://www.jalview.org) and conserved amino acids are shown in blue. Red arrows indicate the catalytic and substrate interacting residues. **b**, Pattern of hydrolysis of manno-oligosaccharides by *Ri*GH113. Enzyme assays were performed overnight. **c**, Pattern of hydrolysis of M_5 by *Ri*GH113. Samples were withdrawn at the indicated time, enzymes were inactivated using 100 mM NaOH and the hydrolysates were analyzed by HPAEC-PAD. **d**, Reaction products of disaccharides catalyzed by *Ri*GH113. **e**, Hydrolysis of Gal_1M_2 plus M_3 and Gal_2M_5 by *Ri*GH113. The reaction products were analyzed by HPAEC-PAD. Gal_1M_4, indicates galactosylmannotetraose. In all panels, control reactions without enzyme did not yield products.

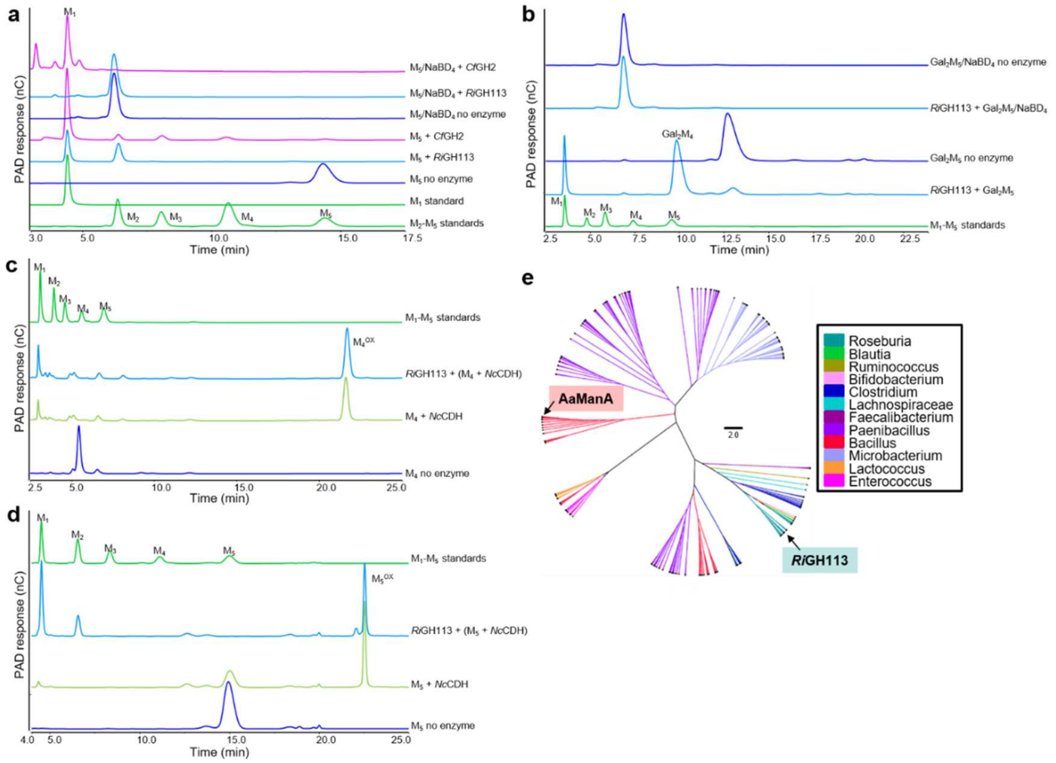

Supplementary Figure 10. Phylogeny and inhibition of *Ri*GH113 activity when blocking the reducing end of various manno-oligosaccharides. a, Analysis of *Ri*GH113 activity on native and reduced M_5. A commercial *Cellulomonas fimi* GH2 β-mannosidase (*Cf*GH2, Megazyme), which acts from the non-reducing end sugar, was used as control. M_5 was pretreated with $NaBD_4$ to reduce the reducing (downstream) end mannose unit into mannitol. *Cf*GH2 fully hydrolyzes the reduced mannopentamer, whereas *Ri*GH113 is not able to hydrolyze it. Note that the reduced oligosaccharide has a considerably shorter retention time than its corresponding native oligosaccharide. **b**, HPAEC-PAD analysis of the activity of *Ri*GH113 on native and reduced Gal_2Man_5. Gal_2Man_5 was treated with $NaBD_4$ before addition of *Ri*GH113. **c**, *Ri*GH113 hydrolysis of native and oxidized M_4 and **d**, M_5 analyzed with HPAEC-PAD. The substrates were pretreated with a *Neurospora crassa* cellobiose dehydrogenase (*Nc*CDH), which oxidizes the reducing end monosaccharide unit into the corresponding lactone/aldonic acid, resulting in a considerably longer retention time than its corresponding native oligosaccharide. *Ri*GH113 is not able to hydrolyze the oxidized oligosaccharide. **e**, Phylogenetic tree of *Ri*GH113 and homologs identified by a Blast search against the NCBI non-redundant protein database. Sequences with coverage ≥86% and identity ≥40% were selected. The resulting 298 sequences were aligned using Muscle. A phylogenetic tree was generated using the maximum likelihood algorithm with 500 bootstrap repetitions in Mega 7 (http://megasoftware.net/). The tree was visualized using Figtree (http://tree.bio.ed.ac.uk/software/figtree). *Ri*GH113 and AaManA are indicated with a black arrow. All sequences were identified as members of the Firmicutes phylum with the exception of those belonging to Microbacterium (Actinobacteria).

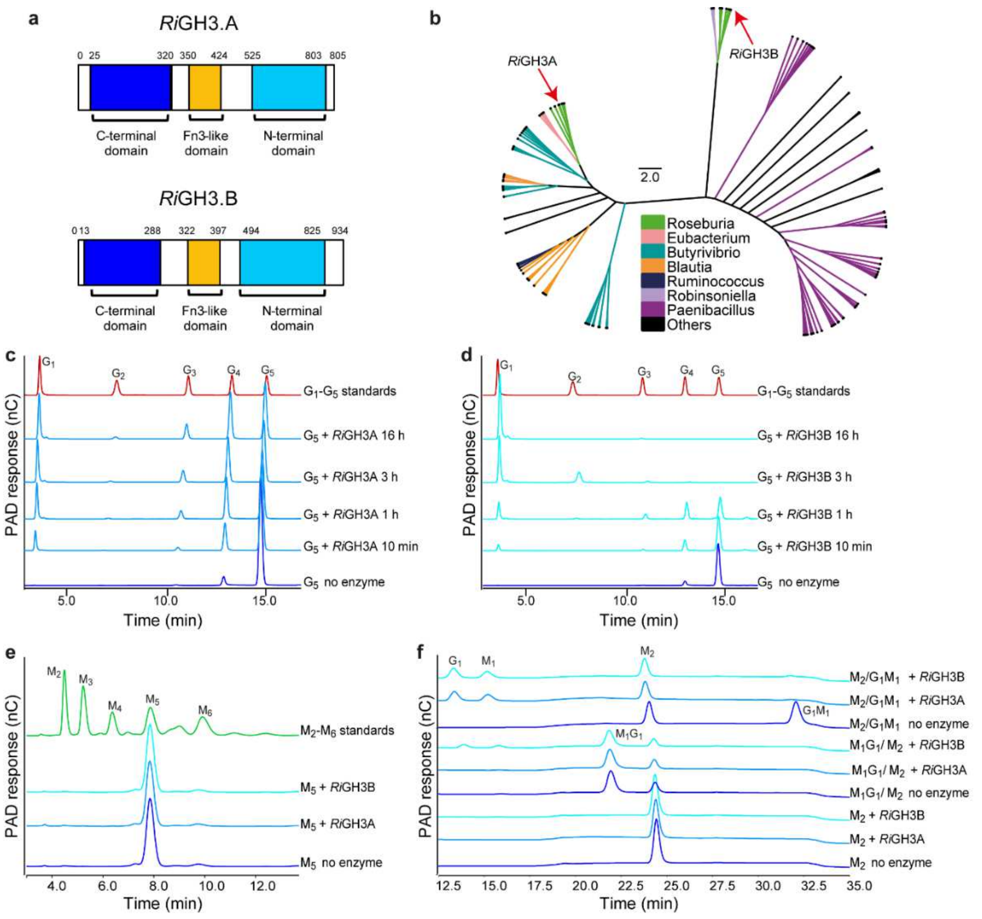

Supplementary Figure 11. Phylogeny of *Ri*GH3A, *Ri*GH3B and homologs and β-glucosidases degradation of different oligosaccharides over time. a, Domain organization of *Ri*GH3A and *Ri*GH3B. Similar to other β-glucosidases from Clostridiales[4], the N- and C-terminal modules in both *Ri*GH3A and *Ri*GH3B are arranged in reverse sequence. **b**, Phylogenetic tree of *Ri*GH3A, *Ri*GH3B and homologs identified by BlastP search against the NCBI non-redundant protein database. Sequences with coverage <86% and identity <40% were removed. The resulting 183 sequences were aligned using Muscle. A phylogenetic tree was generated using the maximum likelihood algorithm with 500 bootstrap repetitions in Mega 7 (http://megasoftware.net/). The tree was visualized using Figtree (http://tree.bio.ed.ac.uk/software/figtree). *Ri*GH3A and *Ri*GH3B are indicated with a red arrow. Despite an apparent redundancy in structure and biochemical function, the two β-glucosidases appear to have diverged significantly, sharing only 40% identity at the amino acid level. Time course analysis of enzymatic reactions containing **c**, *Ri*GH3A and **d**, *Ri*GH3B. Equal amount of enzymes (10 nM) were used. Aliquots were taken at the indicated time points and the enzymes were inactivated by adding NaOH to 100 mM. **e**, HPAEC-PAD analysis of M_5 incubated overnight in the absence or presence of *Ri*GH3A and *Ri*GH3B, showing that neither of them are active on this substrate. **f**, Activity of the β-glucosidases *Ri*GH3A and *Ri*GH3B on disaccharides.

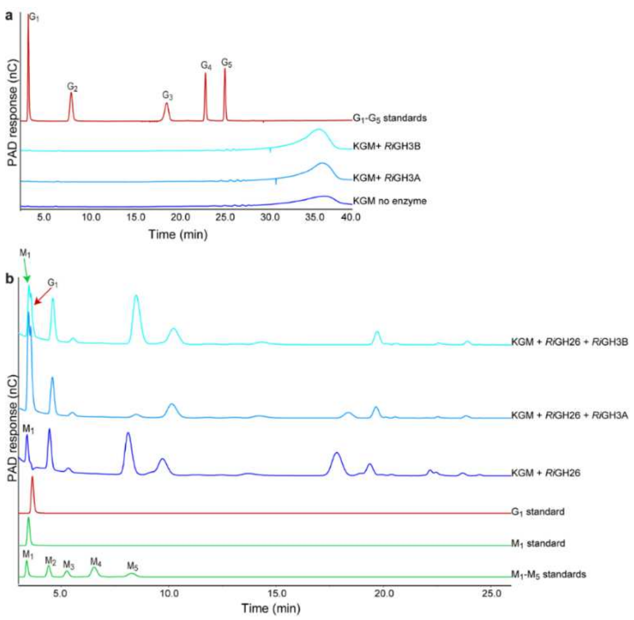

Supplementary Figure 12. *Ri*GH3A and *Ri*GH3B activity on glucomannan. **a**, HPAEC-PAD analysis of *Ri*GH3A or *Ri*GH3B activity on KGM. **b**, Reaction products of *Ri*GH26-digested KGM catalyzed by *Ri*GH3A or *Ri*GH3B.

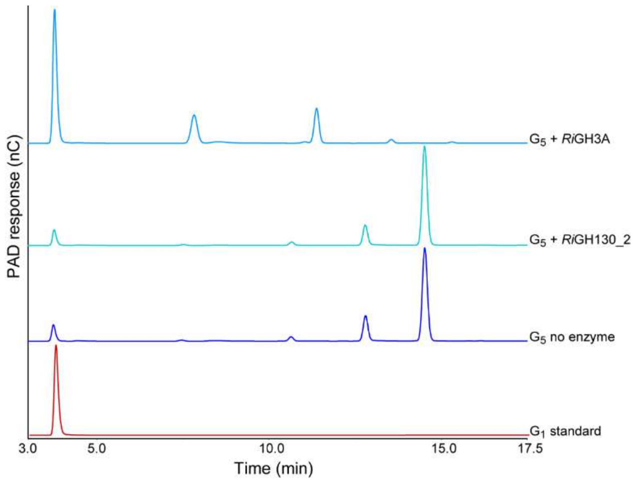

Supplementary Figure 13. Control reaction to check for the activity of *Ri*GH130_2 on cello-oligosaccharides. HPAEC-PAD analysis of G_5 incubated at standard assay conditions in the absence or presence of *Ri*GH130_2. A reaction with the β-glucosidase *Ri*GH3A was used as a control. Only the incubation of *Ri*GH3A with G_5 released a reaction product.

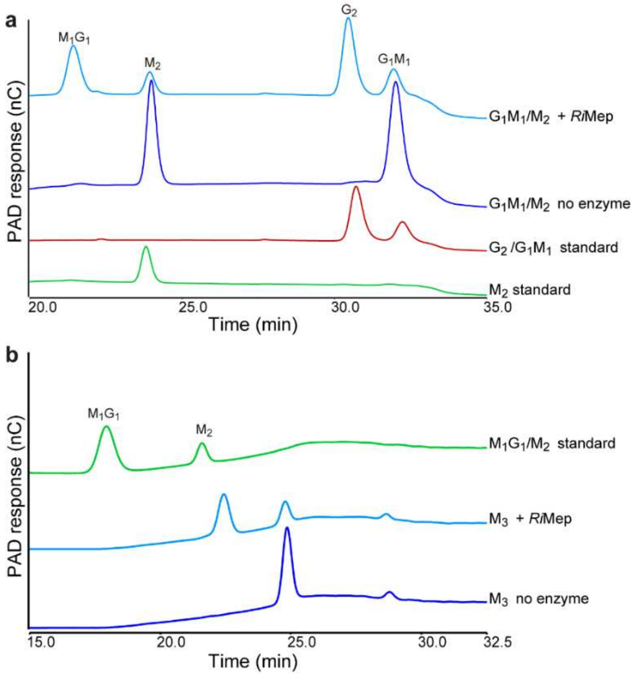

Supplementary Figure 14. HPAEC chromatograms to verify the activity of *Ri*Mep on manno-oligosaccharides. *Ri*Mep was incubated with either **a**, disaccharides or **b**, M_3. *Ri*Mep exhibited epimerization activity towards G_2 and the following manno-oligosaccharides: M_1G_1, G_1M_1, M_3.

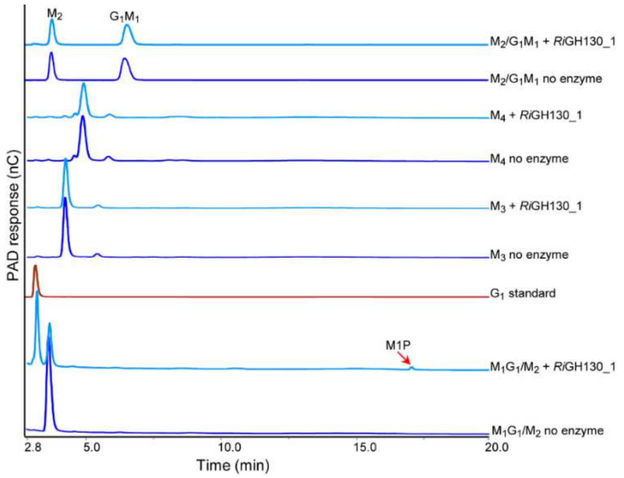

Supplementary Figure 15. Control reactions to verify that *Ri*GH130_1 is active only on M_1G_1. HPAEC-PAD analysis of M_4, M_3, a mixture of M_2 and either M_1G_1 or G_1M_1 incubated under standard assay conditions in the absence or presence of *Ri*GH130_1. The reaction products were subjected to HPAEC and identified by their co-migration with known standards. In this elution method, M_1G_1 co-eluted with M_2.

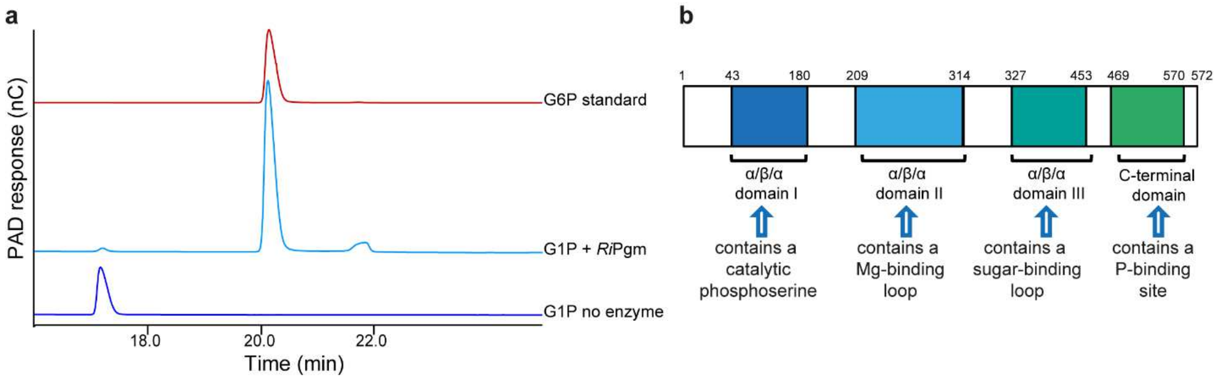

Supplementary Figure 16. Activity on G1P and domain mapping of *Ri*Pgm a, HPAEC-PAD trace showing the activity of *Ri*Pgm on G1P. G1P was either untreated (G1P no enzyme) or treated with *Ri*Pgm. The G6P produced by *Ri*Pgm was identified by co-migration with the G6P standard. **b**, Domain organization of *Ri*Pgm. As observed for other phosphohexomutases[5], the *Ri*Pgm protein structure is modular, featuring four domains. The catalytic domain spans from residue 43 to 180 and harbors a conserved active site. Downstream to the catalytic core domain are three additional domains: domain II (residues 209-314) contains a conserved magnesium (Mg) -binding loop; domain III (residues 327-453) contains the sugar-binding loop that participates in recognizing the two binding orientations of the 1- and 6-phospho-sugars; and the C-terminal domain IV (residues 469-570) harbors a phosphate (P) -binding site that is required for accommodating the incoming phospho-sugar substrate.

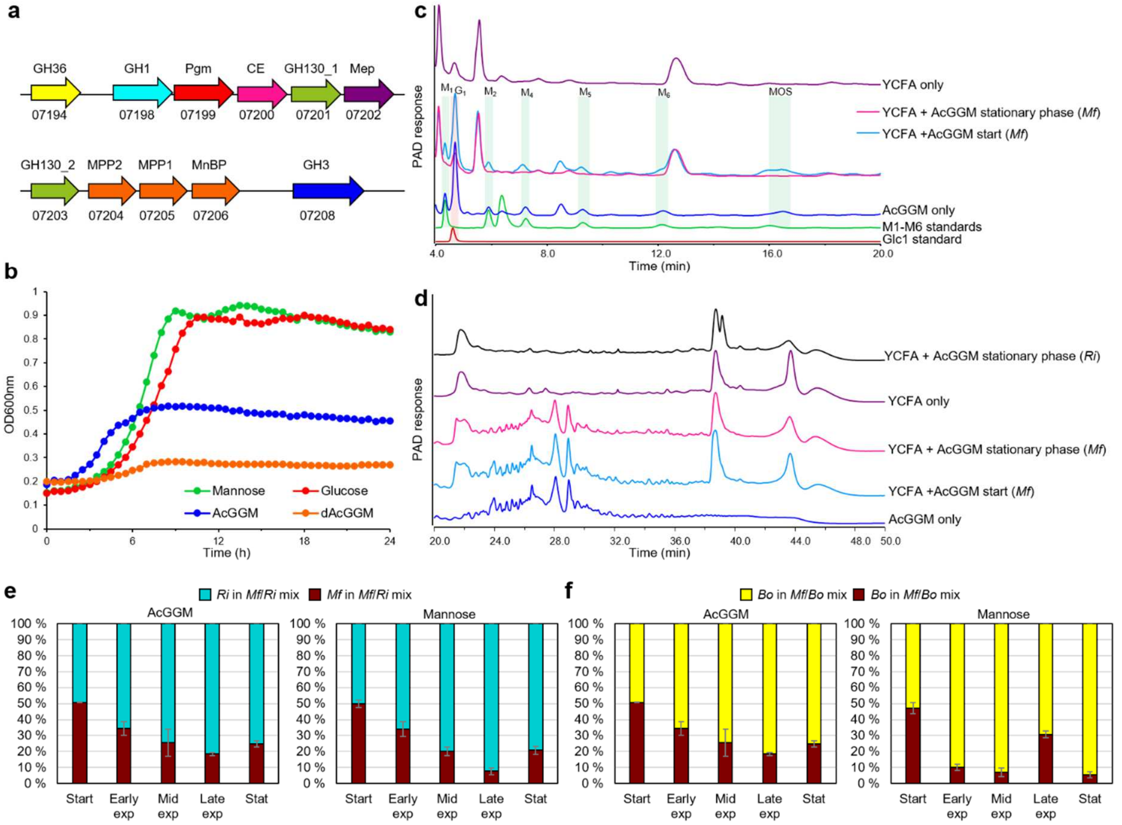

Supplementary Figure 17. *M. formatexigens* β-manno-oligosaccharides utilization cluster, growth profile, glycan consumption and competition experiments. a, Schematic presentation of the putative *M. formatexigens* DSM 14469 β-manno-oligosaccharides degradation locus. Genes are indicated by their locus tag (BRYFOR_XXXXX is abbreviated with the last numbers after the underscore), assignment to CAZyme families and other predicted functions and they are colored according to Fig. 2d. None of the genes has a signal peptide as predicted by the SignalP 4.1 server, suggesting an intracellular location. Absence of a predicted extracellular endomannanase suggests that *M. formatexigens* has no hydrolytic capabilities toward polymeric mannans. **b**, *M. formatexigens* growth on YCFA containing mannose (green), glucose (red), AcGGM (blue) or dyalized AcGGM (dAcGGM, dyalized with a Spectra/Por dyalisis tubing 1KDa MW, Repligen, USA) at 0.5% (w/v). The growth experiment was conducted in 200 µl cultures in 96-well microtiter plates incubated at 37 ºC in anaerobic conditions. Growth was assessed by measuring the absorbance at 600 nm (OD600 nm) at 15 min intervals using a Powerwave HT absorbance reader (Biotek Instruments, Winooski, VT). HPAEC-PAD traces showing **c**, mono- and oligo-saccharides and **d**, polysaccharides contained in the YCFA supplemented with AcGGM before (turquoise) and after (pink) fermentation with *M. formatexigens* (*Mf*). In panel **c**, MOS indicates manno-oligosaccharides. In panel **d**, samples were chromatographed with the spent supernatant of *R. intestinalis* (*Ri*) after overnight growth on YCFA supplemented with AcGGM at 0.5% (w/v). The data displayed are examples from three biological replicates. The traces show that *M. formatexigens* can utilize oligosaccharides (in panel c), but not the polymeric fraction (in panel d). Relative strain abundance during growth of co-cultures of *M. formatexigens* (*Mf*, dark red) and either **e**, *R. intestinalis* (*Ri*, turquoise) or **f**, *B. ovatus* (*Bo*, yellow) on AcGGM or mannose . The ratio of strains in the co-culture was determined by qPCR using primers described in[6]. Histogram bars show the mean of a biological triplicate. Error bars represent s.d.

SUPPLEMENTARY REFERENCES

1 Biely, P. et al. Mode of action of acetylxylan esterases on acetyl glucuronoxylan and acetylated oligosaccharides generated by a GH10 endoxylanase. *Biochim. Biophys. Acta* **1830**, 5075-5086 (2013).

2 Larsen, N. et al. Gut microbiota in human adults with type 2 diabetes differs from non-diabetic adults. *PLoS One* https://doi.org/10.1371/journal.pone.0009085 (2010).

3 Yu, Y., Lee, C., Kim, J. & Hwang, S. Group-specific primer and probe sets to detect methanogenic communities using quantitative real-time polymerase chain reaction. *Biotechnol. Bioeng.* **89**, 670-679 (2005).

4 Harvey, A. J., Hrmova, M., De Gori, R., Varghese, J. N. & Fincher, G. B. Comparative modeling of the three-dimensional structures of family 3 glycoside hydrolases. *Proteins* **41**, 257-269 (2000).

5 Shackelford, G. S., Regni, C. A. & Beamer, L. J. Evolutionary trace analysis of the alpha-D-phosphohexomutase superfamily. *Protein Sci.* **13**, 2130-2138 (2004).

6 Desai, M. S. et al. A dietary fiber-deprived gut microbiota degrades the colonic mucus barrier and enhances pathogen susceptibility. *Cell* **167**, 1339-1353 (2016).
