# Supplementary material for: The human gut Firmicute Roseburia intestinalis is a primary degrader of dietary β-mannans
Source: Nat Commun. 2019 Feb 22;10:905. doi: 10.1038/s41467-019-08812-y (PMC6385246; doi:10.1038/s41467-019-08812-y)
Supplement: Supplementary file 3 — Description of Additional Supplementary Files [file 41467_2019_8812_MOESM3_ESM.docx]

**Description of Supplementary Files**

**File Name:** Supplementary Data 1

**Description:** List of putative CAZy domain-encoding genes upregulated on AcGGM and KGM.

**File Name:** Supplementary Data 2

**Description:** List of R. intestinalis proteins upregulated when grown on AcGGM versus Glc.
