# Supplementary material for: The human gut Firmicute Roseburia intestinalis is a primary degrader of dietary β-mannans
Source: Nat Commun. 2019 Feb 22;10:905. doi: 10.1038/s41467-019-08812-y (PMC6385246; doi:10.1038/s41467-019-08812-y)
Supplement: Supplementary file 4 — Supplementary Data 1 [file 41467_2019_8812_MOESM4_ESM.docx]

**Supplementary Data 1. List of putative CAZy domain-encoding genes upregulated on AcGGM and KGM.** RNA-seq results of upregulation under β-mannans induction normalized to a glucose background are shown with Log_2_-fold change values in bold type. Experiments were performed in three biological replicates. Locus tag numbers ROSINTL182_XXXXX are abbreviated with the last numbers after the hyphen. The presence of a predicted Signal Peptide (SP) was determined with SignalP (v.4.1). Upregulation of CAZyme genes associated with the degradation of other polysaccharides such as α-mannan, arabinan, α-rhamnosides and xyloglucan is ascribed to the presence of these components in the YCFA medium.

|  |  |  |  | |  |  | | | **Log_2_-Fold Change** | |
| --- | --- | --- | --- | --- | --- | --- | --- | --- | --- | --- |
| **Locus**  **Tag** | **CAZyme Family** | **Predicted Activity** | | **Annotation** | | | **SP** | **AcGGM** | | **KGM** |
| 07683 | CBM23,CBM27,GH26 | β-Mannanase | | Fibronectin type III domain protein | | | Yes | **5.80** | | **7.25** |
| 05474 | GH130 | β-1,4-Mannooligosaccharide phosphorylase | | Hypothetical protein | | | No | **5.63** | | **5.42** |
| 05475 | GH130 | β-1,4-Mannosylglucose phosphorylase | | Hypothetical protein | | | No | **5.47** | | **5.57** |
| 05476 | GT4 | Epimerase | | N-acylglucosamine 2-epimerase | | | No | **5.41** | | **5.63** |
| 08144 | GH94 | Cellobiose phosphorylase | | N,N'-diacetylchitobiose phosphorylase | | | No | **4.48** | | **3.58** |
| 09616 | GH23, GT61 | Peptidoglycan lyase | | NlpC/P60 family protein | | | No | **4.43** | | **2.60** |
| 08143 | GH94 | Cellobiose phosphorylase | | N,N'-diacetylchitobiose phosphorylase | | | No | **4.31** | | **3.76** |
| 07684 | GH3 | β-Glucosidase | | β-Glucosidase | | | No | **4.21** | | **4.28** |
| 05473 | CE2 | Acetyl xylan esterase | | Hypothetical protein | | | No | **4.21** | | **4.10** |
| 07685 | GH3 | β-Glucosidase | | β-Glucosidase | | | No | **4.13** | | **4.32** |
| 07158 | GH23 | Peptidoglycan lyase | | Transglycosylase SLT domain protein | | | No | **4.13** | | **2.99** |
| 08193 | GH43 | α-L-Arabinofuranosidase | | Arabinofuranosidase | | | No | **3.88** | | **3.58** |
| 06992 | GH23 | Peptidoglycan lyase | | Putative lipoprotein | | | Yes | **3.83** | | **2.18** |
| 08195 | CBM35,GH115 | Xylan α-1,2-glucuronidase | | Hypothetical protein | | | No | **3.74** | | **3.40** |
| 08196 | GH8 | Reducing-end-xylose releasing exo-oligoxylanase | | Glycosyl hydrolase family 8 | | | No | **3.69** | | **3.54** |
| 07032 | CE1 | Esterase | | Hydrolase, alpha/beta domain protein | | | No | **3.69** | | **1.81** |
| 05471 | CE2, CE3 | Acetyl xylan esterase | | GDSL-like protein | | | No | **3.55** | | **3.47** |
| 06494 | CBM22, CBM9,GH10 | Endo-1,4-β-xylanase | | Endo-1,4- β -xylanase | | | No | **3.35** | | **2.48** |
| 08399 | GH74 | Endoglucanase | | Candidate Xyloglucanase | | | No | **3.34** | | **2.12** |
| 06334 | GH27 | α-Galactosidase | | α-Galactosidase | | | No | **3.33** | | **2.38** |
| 06340 | GH51,GH43 | β-Xylosidase/α-L-Arabinofuranosidase | | Hypothetical protein | | | No | **3.27** | | **1.80** |
| 09628 | GH3 | β-Glucosidase | | β-Glucosidase | | | No | **3.23** | | **1.83** |
| 05034 | CBM35 | Xylan-binding module | | ABC Transporter | | | No | **3.19** | | **1.87** |
| 06343 | CBM6, GH43 | α-L-Arabinofuranosidase | | Arabinofuranosidase | | | No | **3.07** | | **1.98** |
| 06338 | CBM22, GH10 | Endo-1,4-β-xylanase | | Endo-1,4- β -xylanase | | | No | **3.06** | | 1.44 |
| 08567 | GT41 | UDP-GlcNAc:peptide Acetylglucosaminyltransferase | | Tetratricopeptide repeat protein | | | Yes | **3.02** | | **4.02** |
| 09626 | GH42, GH5 | Cellulase/β-galactosidase | | Hypothetical protein | | | No | **3.01** | | **2.02** |
| 08606 | CBM50 | Peptidoglycan-binding module | | LysM domain protein | | | No | **2.85** | | **2.06** |
| 05969 | GT2 | Glycosyltransferase, group 2 family | | Glycosyltransferase, group 2 family | | | No | **2.82** | | 0.68 |
| 09282 | GT83 | 4-amino-4-deoxy-β-L-Arabinosyltransferase | | Hypothetical protein | | | No | **2.76** | | 0.97 |
|  |  |  | |  | | |  | **Log_2_-Fold Change** | | |
| **Locus**  **Tag** | **CAZyme Family** | **Predicted Activity** | | **Annotation** | | | **SP** | **AcGGM** | | **KGM** |
| 06337 | GH43 | β-Xylosidase/α-L-Arabinofuranosidase | | β-Xylosidase/α-L-Arabinofuranosidase | | | No | **2.75** | | **1.54** |
| 06342 | CBM48, CE1 | Acetyl xylan esterase | | Putative xylanase | | | No | **2.67** | | 1.47 |
| 06341 | GH43 | α-L-Arabinofuranosidase | | α-L-Arabinofuranosidase | | | No | **2.67** | | **1.88** |
| 07678 | CBM61, GH53 | endo-β-1,4-galactanase | | Endo-β-1,4-galactanase | | | No | **2.65** | | 1.41 |
| 08358 | CE10, CE7 | Acetyl xylan esterase/arylesterase | | Lysophospholipase | | | No | **2.62** | | 1.02 |
| 09560 | GH78 | α-L-Rhamnosidase | | α-L-Rhamnosidase | | | No | **2.56** | | 1.35 |
| 05470^a^ | GH1 | β-Mannosidase/β-Glucosidase | | Glycosyl hydrolase, family 1 | | | No | **2.56** | | **2.09** |
| 06940 | CBM37 | Xylan-binding module | | Repeat protein | | | No | **2.53** | | 1.17 |
| 06332 | GH32, GH43 | β-2,1-Fructosidase/α-L-Arabinofuranosidase | | α-L-Arabinofuranosidase | | | No | **2.52** | | 1.41 |
| 08710 | GH94 | Cellobiose phosphorylase | | Cellobiose phosphorylase | | | No | **2.50** | | 1.42 |
| 05469^a^ | GH1 | Mannose-6-phosphate isomerase | | Mannose-6-phosphate isomerase | | | No | **2.38** | | **2.15** |
| 06339 | GH10 | Endo-1,4-β-xylanase | | Endo-1,4-β-xylanase | | | Yes | **2.37** | | 0.51 |
| 05892 | GH43 | β-Xylosidase/α-L-Arabinofuranosidase | | β-Xylosidase/α-L-Arabinofuranosidase | | | No | **2.32** | | 1.37 |
| 09562 | GH94 | Cellobiose phosphorylase | | N,N'-diacetylchitobiose phosphorylase | | | No | **2.31** | | 1.17 |
| 08400 | GH31 | α-Glucosidase | | α-Glucosidase | | | No | **2.27** | | 1.40 |
| 06335 | GH43 | β-Xylosidase/α-L-Arabinofuranosidase | | β-Xylosidase/α-L-Arabinofuranosidase | | | No | **2.26** | | 0.87 |
| 05893 | GH43 | β-Xylosidase/α-L-Arabinofuranosidase | | β-Xylosidase | | | No | **2.22** | | 1.36 |
| 05114 | GH115 | Xylan α-1,2-glucuronidase | | Hypothetical protein | | | No | **2.19** | | 0.85 |
| 08711 | GH94 | Cellobiose phosphorylase | | N,N'-diacetylchitobiose phosphorylase | | | No | **2.16** | | 0.97 |
| 09559 | GH3 | β-Glucosidase | | β-Glucosidase | | | No | **2.15** | | 1.32 |
| 06336 | GH95 | α-L-Fucosidase | | Hypothetical protein | | | No | **2.11** | | 1.00 |
| 09625 | GH94 | Cellobiose phosphorylase | | N,N'-diacetylchitobiose phosphorylase | | | No | **2.09** | | 0.96 |
| 09492 | GH2 | β-Galactosidase | | β-Galactosidase | | | No | **2.09** | | 1.03 |
| 09554 | GH35 | β-Galactosidase | | β-Galactosidase | | | No | **1.99** | | 0.65 |
| 08556 | GH5 | Endo-β-1,4-glucanase/Cellulase | | Endoglucanase A | | | No | **1.99** | | **2.88** |
| 09556 | GH3 | β-Glucosidase | | β-Glucosidase | | | No | **1.92** | | 0.97 |
| 05638 | GH35 | β-Galactosidase | | Glycosyl hydrolase, family 35 | | | No | **1.86** | | 0.86 |
| 08720 | GH38 | α-Mannosidase | | α-Mannosidase | | | No | **1.84** | | 0.69 |
| 05483 | GH113 | β-mannanase | | Hypothetical protein | | | No | **1.80** | | 0.68 |
| 05574 | GH119 | α-Amylase | | Hypothetical protein | | | No | **1.77** | | 0.74 |
|  |  |  | |  | | |  | **Log_2_-Fold Change** | | |
| **Locus**  **Tag** | **CAZyme Family** | **Predicted Activity** | | **Annotation** | | | **SP** | **AcGGM** | | **KGM** |
| 05891 | CE12 | Acetyl xylan esterase | | Hypothetical protein | | | No | **1.77** | | 0.73 |
| 08203 | GH42 | β-Galactosidase/α-L-Arabinopyranosidase | | β-Galactosidase | | | No | **1.77** | | 0.92 |
| 06331 | GH43 | β-Xylosidase/α-L-Arabinofuranosidase | | β-Xylosidase/α-L-Arabinofuranosidase | | | No | **1.70** | | 0.84 |
| 09491 | GH2 | β-Galactosidase | | β-Galactosidase | | | No | **1.70** | | 0.79 |
| 09478 | GH73 | Endo-β-N-acetylglucosaminidase | | Hypothetical protein | | | No | **1.67** | | 0.54 |
| 05481 | GH36 | α-Galactosidase | | α-Galactosidase | | | No | **1.64** | | 1.26 |
| 08825 | GH13 | α-Amylase | | α-Amylase | | | No | **1.64** | | 0.84 |
| 08557 | GH32, GH43 | Fructan β-2,1-fructosidase/α-L-Arabinofuranosidase | | Hypothetical protein | | | No | **1.63** | | **2.51** |
| 08719 | GH2 | β-Galactosidase | | β-Galactosidase | | | No | **1.61** | | 0.58 |
| 07340 | CE1 | Esterase | | Hydrolase | | | No | **1.60** | | 0.78 |
| 09481 | GH24 | Endo-β-N-acetylglucosaminidase | | Hypothetical protein | | | No | **1.59** | | 0.47 |

^a^Loci ROSINTL182_05469 and ROSINTL182_05470 are annotated as two proteins of 522 and 232 amino acids, respectively. However, we corrected sequence ambiguities at the 3’-end of

ROSINTL182_05470 and determined the absence of a stop codon in between the two genes.
