# Supplementary material for: The human gut Firmicute Roseburia intestinalis is a primary degrader of dietary β-mannans
Source: Nat Commun. 2019 Feb 22;10:905. doi: 10.1038/s41467-019-08812-y (PMC6385246; doi:10.1038/s41467-019-08812-y)
Supplement: Supplementary file 5 — Supplementary Data 2 [file 41467_2019_8812_MOESM5_ESM.docx]

**Supplementary Data 2. List of *R. intestinalis* proteins upregulated when grown on AcGGM versus Glc.** Proteins are annotated with the UniProt accession numbers and putative function. Locus tag numbers ROSINTL182_XXXXX are abbreviated with the last numbers after the hyphen. Values given for the carbon sources are Log_2_ of MaxLFQ intensities, median of three biological replicates. Proteins involved in β-mannan catabolism are in bold.

|  |  |  | **Median Log_2_ LFQ Intensities** | |  |  |
| --- | --- | --- | --- | --- | --- | --- |
| **UniProt ID** | **Locus tag** | **CAZy Family/Predicted function** | **Glc** | **AcGGM** | **Diff.^a^** | **Peptide count** |
| C7GAP5 | 06975 | Pyridine nucleotide-disulfide oxidoreductase | 19.14 | 28.71 | 9.57 | 8 |
| **C7G6G5** | **05478** | **MPP1, ABC transporter, permease protein** | **17.82** | **26.92** | **9.10** | **9** |
| **C7GCP5** | **07683** | **GH26 / β-mannosidase** | **20.69** | **29.25** | **8.56** | **85** |
| **C7G6G2** | **05475** | **GH130_1 / Mannosyl-glucose phosphorylase** | **21.76** | **29.58** | **7.82** | **41** |
| **C7G6G4** | **05477** | **MPP2, ABC transporter, permease protein** | **18.47** | **25.97** | **7.50** | **9** |
| **C7G6G3** | **05476** | **Mep, Epimerase** | **16.63** | **24.02** | **7.39** | **31** |
| C7G9B3 | 06492 | GH1 / Arabinogalactan endo-galactanase | 17.49 | 24.13 | 6.64 | 9 |
| **C7G6G1** | **05474** | **GH130_2/ Mannoside phosphorylase** | **23.91** | **30.17** | **6.26** | **28** |
| **C7G6G6** | **05479** | **ABC transporter, mannan-binding protein** | **24.84** | **30.83** | **5.98** | **21** |
| C7GE35 | 08187 | Hypothetical protein | 21.07 | 27.01 | 5.95 | 40 |
| **C7GCP6** | **07684** | **GH3B / β -glucosidase** | **19.37** | **25.19** | **5.82** | **32** |
| **C7GCP7** | **07685** | **GH3A / β -glucosidase** | **20.70** | **26.45** | **5.75** | **48** |
| C7GE41 | 08193 | GH43 / α-arabinofuranosidase | 18.17 | 23.82 | 5.65 | 28 |
| C7GF65 | 08570 | ABC transporter, solute-binding protein | 18.87 | 24.44 | 5.56 | 10 |
| C7GF66 | 08571 | Hypothetical protein | 18.33 | 23.89 | 5.56 | 17 |
| C7GER5 | 08417 | Maturation rSAM protein, HydE | 17.50 | 22.76 | 5.26 | 4 |
| C7GF51 | 08556 | GH1 / Endoglucanase | 18.59 | 23.57 | 4.97 | 5 |
| **C7G6G0** | **05473** | **Carbohydrate esterase, CE2** | **19.06** | **24.02** | **4.96** | **16** |
| C7GF52 | 08557 | Hypothetical protein | 19.32 | 24.18 | 4.86 | 18 |
| C7GGA9 | 08977 | Sugar-binding domain protein | 18.78 | 23.58 | 4.80 | 5 |
| **C7G6F8** | **05471** | **Carbohydrate esterase, CEX** | **21.81** | **26.53** | **4.72** | **29** |
| C7GE44 | 08196 | GH8 /reducing-end xylose releasing oligoxylanase | 18.12 | 22.83 | 4.71 | 12 |
| C7GE47 | 08199 | ABC transporter, xylan-binding protein | 21.72 | 26.36 | 4.64 | 27 |
| C7GE46 | 08198 | ABC transporter, permease protein | 19.22 | 23.84 | 4.62 | 2 |
| C7GDZ0 | 08142 | Transcriptional regulator, LacI family | 19.64 | 24.12 | 4.49 | 12 |
| C7G8A2 | 06126 | Enolase | 13.92 | 18.33 | 4.41 | 30 |
| C7GB09 | 07093 | Riboflavin biosynthesis protein, RibH | 20.58 | 24.99 | 4.41 | 15 |
| C7GE51 | 08203 | β-galactosidase | 20.23 | 24.63 | 4.40 | 23 |
| C7GCG9 | 07606 | Hypothetical protein | 18.15 | 22.53 | 4.38 | 4 |
| **C7G6H0** | **05483** | **GH113** | **19.57** | **23.93** | **4.36** | **13** |
| C7G9G1 | 06540 | Hypothetical protein | 19.35 | 23.39 | 4.04 | 7 |
| C7GF55 | 08560 | Hypothetical protein | 23.21 | 27.24 | 4.03 | 14 |
| C7GD06 | 07806 | 30S ribosomal protein S10 | 21.10 | 25.02 | 3.92 | 7 |
| C7GCL0 | 07648 | Membrane protein insertase, YidC/Oxa1 family | 16.19 | 20.07 | 3.88 | 14 |
| C7G9Z7 | 06727 | TRAP transporter solute receptor, DctP family | 19.40 | 23.25 | 3.85 | 8 |
| C7G830 | 06054 | 30S ribosomal protein | 19.86 | 23.66 | 3.80 | 4 |
| C7GE73 | 08224 | Endonuclease III | 16.77 | 20.53 | 3.76 | 4 |
| C7GEV7 | 08459 | Signal recognition particle protein | 17.84 | 21.59 | 3.75 | 29 |
| C7GH91 | 09312 | Putative translation elongation factor G | 20.71 | 24.45 | 3.74 | 41 |
|  |  |  | **Median Log_2_ LFQ Intensities** | |  |  |
| **UniProt ID** | **Locus tag** | **CAZy Family/Predicted function** | **Glc** | **AcGGM** | **Diff.^a^** | **Peptide count** |
| C7G5M9 | 05187 | IS66 family element, transposase | 17.74 | 21.47 | 3.73 | 9 |
| C7GHZ4 | 09566 | β-lactamase | 19.45 | 23.11 | 3.66 | 9 |
| C7GF62 | 08567 | Tetratricopeptide repeat protein | 18.75 | 22.34 | 3.59 | 5 |
| C7GFK0 | 08707 | Hypothetical protein | 21.04 | 24.62 | 3.58 | 7 |
| C7G9U2 | 06672 | GH38 / α-mannosidase | 20.98 | 24.51 | 3.54 | 42 |
| C7GDY7 | 08139 | Hypothetical protein | 21.74 | 25.27 | 3.53 | 9 |
| C7G7D3 | 05800 | ABC transporter, ATP-binding protein | 18.03 | 21.52 | 3.49 | 27 |
| C7GB06 | 07090 | Riboflavin biosynthesis protein, RibD | 17.66 | 21.12 | 3.46 | 8 |
| C7GD03 | 07803 | 50S ribosomal protein L23 | 21.22 | 24.67 | 3.45 | 8 |
| C7G702 | 05669 | RelA/SpoT family protein | 21.79 | 25.15 | 3.35 | 32 |
| C7GCZ6 | 07796 | 30S ribosomal protein S17 | 21.65 | 24.98 | 3.33 | 8 |
| C7GD85 | 07886 | ABC transporter, ATP-binding protein | 19.08 | 22.39 | 3.32 | 41 |
| C7GCG3 | 07600 | ABC transporter, solute-binding protein | 20.18 | 23.44 | 3.26 | 16 |
| C7G7L4 | 05888 | ABC transporter, glycerol-3-phosphate transport system | 18.66 | 21.89 | 3.24 | 21 |
| **C7G6G9** | **05482** | **Pgm, Phosphomannomutase** | **21.70** | **24.84** | **3.14** | **62** |
| C7GDL8 | 08019 | Transketolase, Pentose phosphate pathway | 18.99 | 22.12 | 3.14 | 4 |
| C7GE45 | 08197 | ABC transporter, permease protein | 19.66 | 22.78 | 3.12 | 6 |
| C7GB02 | 07086 | Response regulator receiver domain protein | 18.91 | 21.97 | 3.06 | 10 |
| C7GDY8 | 08140 | Hypothetical protein | 22.35 | 25.39 | 3.04 | 3 |
| C7GDI0 | 07981 | Isoprenoid synthesis protein, IspH | 18.51 | 21.54 | 3.03 | 6 |
| C7GDW1 | 08113 | Hsp20/alpha crystallin family protein | 17.12 | 20.12 | 3.00 | 3 |
| C7GF11 | 08514 | Hypothetical protein | 17.75 | 20.75 | 3.00 | 4 |
| C7G6K9 | 05523 | Polyphosphate:AMP phosphotransferase | 20.68 | 23.66 | 2.98 | 17 |
| C7GBV3 | 07388 | Flagellar basal body protein | 21.61 | 24.58 | 2.97 | 22 |
| C7GCJ0 | 07628 | Pantothenate synthetase | 23.69 | 26.66 | 2.96 | 33 |
| C7GDZ1 | 08143 | Glycosyltransferase family 36 | 22.95 | 25.91 | 2.96 | 18 |
| C7G5E4 | 05102 | Aminopeptidase | 20.95 | 23.91 | 2.95 | 38 |
| C7GHF0 | 09371 | GH31 / putative α-xylosidase | 21.55 | 24.50 | 2.95 | 21 |
| C7GCI9 | 07627 | Aspartate 1-decarboxylase | 21.25 | 24.17 | 2.92 | 7 |
| C7G750 | 05717 | Hypothetical protein | 18.61 | 21.51 | 2.90 | 6 |
| C7GDM1 | 08023 | L-arabinose isomerase | 18.61 | 21.51 | 2.90 | 13 |
| C7G674 | 05384 | Putative guanine deaminase | 19.64 | 22.53 | 2.90 | 18 |
| C7G5X1 | 05280 | VanW-like protein | 21.63 | 24.50 | 2.87 | 12 |
| C7GE21 | 08173 | DNA helicase | 20.65 | 23.51 | 2.86 | 37 |
| C7G7T5 | 05959 | Hydrolase | 17.63 | 20.49 | 2.86 | 8 |
| C7G5L1 | 05169 | Malonyl CoA-acyl carrier protein transacylase | 19.33 | 22.19 | 2.86 | 26 |
| C7GFZ7 | 08854 | Translation initiation factor IF-3 | 22.48 | 25.25 | 2.77 | 11 |
| C7GCJ1 | 07629 | 3-methyl-2-oxobutanoate hydroxymethyltransferase | 21.62 | 24.38 | 2.77 | 22 |
| C7GDL7 | 08018 | Transketolase, Pentose phosphate pathway | 21.22 | 23.98 | 2.76 | 5 |
| C7G9C2 | 06501 | Hypothetical α-amylase | 23.76 | 26.51 | 2.76 | 21 |
| C7GDN0 | 08031 | Hypothetical protein | 23.37 | 26.10 | 2.73 | 12 |
| C7GDZ2 | 08144 | Hypothetical protein | 24.69 | 27.42 | 2.73 | 45 |
| C7G6R3 | 05578 | Hypothetical protein | 20.52 | 23.25 | 2.72 | 5 |
| C7GCL9 | 07657 | ParB-like protein | 18.18 | 20.88 | 2.71 | 15 |
| C7GEV6 | 08458 | 30S ribosomal protein S16 | 20.87 | 23.58 | 2.70 | 5 |
|  |  |  | **Median Log_2_ LFQ Intensities** | |  |  |
| **UniProt ID** | **Locus tag** | **CAZy Family/Predicted function** | **Glc** | **AcGGM** | **Diff.^a^** | **Peptide count** |
| C7G6Y0 | 05647 | Putative catabolite control protein A | 17.98 | 20.68 | 2.70 | 3 |
| **C7G6F7** | **05470** | **GH1** | **20.38** | **23.03** | **2.65** | **16** |
| C7G7Q2 | 05926 | Ribosomal protein S12 methylthiotransferase, RimO | 21.85 | 24.49 | 2.65 | 21 |
| C7GEF2 | 08304 | von Willebrand factor type A domain protein | 22.12 | 24.77 | 2.65 | 17 |
| C7GE38 | 08190 | ABC transporter, solute-binding protein | 22.38 | 25.01 | 2.63 | 12 |
| C7G8L8 | 06244 | Hypothetical phosphoketolase | 21.25 | 23.86 | 2.60 | 42 |
| C7G7C5 | 05792 | ABC transporter, ATP-binding protein | 18.52 | 21.11 | 2.59 | 16 |
| C7GDS7 | 08079 | EDD domain protein, DegV family | 22.43 | 24.99 | 2.56 | 13 |
| C7GCB6 | 07551 | ABC transporter, ATP-binding protein | 23.30 | 25.82 | 2.53 | 21 |
| C7GE37 | 08189 | ABC transporter, permease protein | 18.96 | 21.48 | 2.52 | 3 |
| C7GCF5 | 07592 | Site-determining protein | 23.12 | 25.64 | 2.52 | 18 |
| C7GC93 | 07528 | Phosphopentomutase | 19.20 | 21.71 | 2.51 | 20 |
| C7GE43 | 08195 | GH115 / α-glucuronidase | 18.06 | 20.56 | 2.50 | 9 |
| C7GDL9 | 08020 | Transketolase, thiamine diphosphate binding domain protein | 21.69 | 24.19 | 2.50 | 5 |
| C7GEF1 | 08303 | Hypothetical protein | 26.54 | 29.02 | 2.48 | 74 |
| C7G9L8 | 06597 | Cell shape determining protein, MreB/Mrl family | 21.14 | 23.61 | 2.48 | 20 |
| C7GAG5 | 06895 | Hypothetical protein | 20.29 | 22.74 | 2.45 | 10 |
| C7G8E5 | 06169 | RNA pseudouridine synthase | 18.41 | 20.83 | 2.42 | 4 |
| C7GCX7 | 07777 | 30S ribosomal protein S11 | 22.91 | 25.31 | 2.40 | 12 |
| C7GDX0 | 08122 | HD domain protein | 18.21 | 20.61 | 2.40 | 11 |
| C7G9H0 | 06549 | Glycerophosphotransferase | 18.30 | 20.69 | 2.39 | 41 |
| C7GGC3 | 08991 | TOBE domain protein | 24.99 | 27.38 | 2.38 | 3 |
| C7G5C9 | 05087 | Hypothetical protein | 18.30 | 20.67 | 2.38 | 4 |
| C7GDQ8 | 08059 | Hypothetical protein | 23.67 | 26.04 | 2.37 | 10 |
| C7GGG3 | 09031 | Signal peptidase I | 17.66 | 20.03 | 2.37 | 5 |
| C7GBY2 | 07417 | ABC transporter, solute-binding protein | 21.47 | 23.82 | 2.35 | 12 |
| C7G9R9 | 06648 | Dipeptidase | 16.74 | 19.09 | 2.35 | 16 |
| C7G7B6 | 05783 | Hypothetical protein | 18.06 | 20.41 | 2.34 | 8 |
| C7GG32 | 08894 | Putative glycogen debranching enzyme | 17.64 | 19.96 | 2.33 | 22 |
| C7GE90 | 08242 | DNA mismatch repair protein, MutS | 20.01 | 22.33 | 2.32 | 14 |
| C7GDQ7 | 08058 | Hypothetical protein | 22.86 | 25.18 | 2.31 | 33 |
| C7GCZ5 | 07795 | 50S ribosomal protein L14 | 24.19 | 26.49 | 2.30 | 13 |
| C7GA10 | 06762 | Cell Wall Hydrolase | 23.34 | 25.64 | 2.30 | 10 |
| C7GDM0 | 08021 | Putative transaldolase | 21.84 | 24.13 | 2.29 | 19 |
| C7GDH7 | 07978 | Leucine Rich Repeat protein | 24.43 | 26.71 | 2.29 | 65 |
| C7GC36 | 07471 | Siphovirus Gp157 | 19.46 | 21.74 | 2.28 | 7 |
| C7GEC8 | 08280 | Hypothetical protein | 20.47 | 22.74 | 2.27 | 11 |
| C7GCF7 | 07594 | Aminotransferase, class I/II | 18.27 | 20.54 | 2.27 | 20 |
| C7G7D2 | 05799 | ABC transporter, ATP-binding protein | 19.97 | 22.21 | 2.24 | 37 |
| C7GD01 | 07801 | 30S ribosomal protein S19 | 25.09 | 27.32 | 2.23 | 16 |
| C7GE34 | 08186 | Hypothetical protein | 19.42 | 21.64 | 2.22 | 4 |
| C7G7X0 | 05994 | POTRA domain protein, FtsQ-type | 17.38 | 19.61 | 2.22 | 2 |
| C7G8I6 | 06212 | Diguanylate cyclase domain protein | 19.56 | 21.78 | 2.22 | 9 |
| C7GF48 | 08551 | tRNA N6-adenosine threonylcarbamoyltransferase | 20.09 | 22.30 | 2.22 | 5 |
| C7GEE4 | 08296 | Hypothetical protein | 21.66 | 23.87 | 2.22 | 4 |
|  |  |  | **Median Log_2_ LFQ Intensities** | |  |  |
| **UniProt ID** | **Locus tag** | **CAZy Family/Predicted function** | **Glc** | **AcGGM** | **Diff.^a^** | **Peptide count** |
| C7GHX4 | 09545 | ATP-dependent metallopeptidase HflB | 18.40 | 20.61 | 2.21 | 10 |
| C7GAT0 | 07010 | Glycerol-3-phosphate responsive antiterminator | 20.58 | 22.77 | 2.19 | 12 |
| C7GDH3 | 07974 | Electron transport complex subunit G | 18.83 | 21.00 | 2.17 | 7 |
| C7GEV1 | 08453 | 50S ribosomal protein L19 | 24.84 | 27.00 | 2.16 | 16 |
| C7GCE8 | 07585 | Uridine kinase | 18.81 | 20.95 | 2.14 | 7 |
| C7G5B8 | 05076 | Penicillin-binding protein | 16.98 | 19.12 | 2.14 | 30 |
| C7GCZ4 | 07794 | 50S ribosomal protein L24 | 18.24 | 20.37 | 2.13 | 3 |
| **C7G6G7** | **05480** | **Transcriptional regulator, LacI family** | **21.38** | **23.49** | **2.11** | **9** |
| C7G956 | 06433 | Transcriptional regulator, MarR family | 18.39 | 20.49 | 2.10 | 2 |
| C7GE31 | 08183 | GH53 / Arabinogalactan β-galactanase | 21.37 | 23.46 | 2.09 | 7 |
| C7GE96 | 08248 | Cell shape determining protein, MreB/Mrl family | 20.38 | 22.45 | 2.07 | 16 |
| C7GBN0 | 07315 | ABC transporter, substrate-binding protein, family 5 | 17.55 | 19.61 | 2.06 | 24 |
| C7GCY9 | 07789 | 50S ribosomal protein L6 | 25.88 | 27.92 | 2.05 | 19 |
| C7GCL6 | 07654 | Putative sensor protein, DegS | 18.71 | 20.73 | 2.02 | 8 |
| C7GAP6 | 06976 | FAD dependent oxidoreductase | 18.83 | 20.84 | 2.01 | 4 |
| C7GGY8 | 09209 | Hypothetical protein | 28.44 | 30.44 | 2.00 | 10 |
| C7GED6 | 08288 | Hypothetical protein | 20.37 | 22.36 | 1.99 | 13 |
| C7G9N1 | 06610 | Hypothetical protein | 19.55 | 21.53 | 1.99 | 4 |
| C7GAG4 | 06894 | Hypothetical protein | 21.57 | 23.49 | 1.92 | 11 |
| C7GCL5 | 07653 | Hypothetical protein | 20.65 | 22.54 | 1.89 | 22 |
| C7G890 | 06114 | Hypothetical protein | 19.63 | 21.51 | 1.88 | 18 |
| C7GBN7 | 07322 | Oxidoreductase, aldo/keto reductase family protein | 19.49 | 21.37 | 1.87 | 15 |
| C7GE32 | 08184 | GH2 / β-galactosidase | 21.72 | 23.57 | 1.85 | 42 |
| C7GAY7 | 07068 | Hypothetical protein | 22.70 | 24.52 | 1.81 | 2 |
| **C7G6G8** | **05481** | **GH36 / α-galactosidase** | **23.85** | **25.62** | **1.77** | **57** |
| C7GCF2 | 07589 | Thiamine biosynthesis pathway, ThiH | 20.16 | 21.92 | 1.77 | 4 |
| C7G5Q2 | 05210 | SPFH/Band 7/PHB domain protein | 19.95 | 21.70 | 1.75 | 28 |
| C7GDL6 | 08017 | Rribulose-5-phosphate 4-epimerase | 22.71 | 24.44 | 1.73 | 9 |
| C7G7K2 | 05876 | Sporulation protein YtfJ | 19.72 | 21.39 | 1.68 | 8 |
| C7GDX7 | 08129 | Tetratricopeptide repeat protein | 20.78 | 22.46 | 1.67 | 20 |
| C7GDM3 | 08024 | Carbohydrate kinase | 19.94 | 21.57 | 1.64 | 7 |
| C7G8K4 | 06230 | Cobalt transport protein | 17.60 | 19.23 | 1.63 | 9 |
| C7GDH8 | 07979 | CheR methyltransferase, glutamate biosynthesis | 20.36 | 21.91 | 1.56 | 31 |
| C7GE91 | 08243 | Thymidylate synthase, ThyX | 20.78 | 22.33 | 1.54 | 21 |

^a^Difference between AcGGM and Glc Log_2_-fold values.
