# Supplementary material for: The human gut Firmicute Roseburia intestinalis is a primary degrader of dietary β-mannans
Source: Nat Commun. 2019 Feb 22;10:905. doi: 10.1038/s41467-019-08812-y (PMC6385246; doi:10.1038/s41467-019-08812-y)
Supplement: Supplementary file 6 — Reporting Summary [file 41467_2019_8812_MOESM6_ESM.pdf]

nature research

Corresponding author(s): Bjørge Westereng

Reporting Summary

Nature Research wishes to improve the reproducibility of the work that we publish. This form provides structure for consistency and transparency in reporting. For further information on Nature Research policies, see Authors & Referees and the Editorial Policy Checklist.

Statistical parameters

When statistical analyses are reported, confirm that the following items are present in the relevant location (e.g. figure legend, table legend, main text, or Methods section).

n/a	Confirmed	
☐	☒	The exact sample size (*n*) for each experimental group/condition, given as a discrete number and unit of measurement
☐	☒	An indication of whether measurements were taken from distinct samples or whether the same sample was measured repeatedly
☐	☒	The statistical test(s) used AND whether they are one- or two-sided<br>*Only common tests should be described solely by name; describe more complex techniques in the Methods section.*
☒	☐	A description of all covariates tested
☒	☐	A description of any assumptions or corrections, such as tests of normality and adjustment for multiple comparisons
☒	☐	A full description of the statistics including central tendency (e.g. means) or other basic estimates (e.g. regression coefficient) AND variation (e.g. standard deviation) or associated estimates of uncertainty (e.g. confidence intervals)
☒	☐	For null hypothesis testing, the test statistic (e.g. *F*, *t*, *r*) with confidence intervals, effect sizes, degrees of freedom and *P* value noted<br>*Give P values as exact values whenever suitable.*
☒	☐	For Bayesian analysis, information on the choice of priors and Markov chain Monte Carlo settings
☒	☐	For hierarchical and complex designs, identification of the appropriate level for tests and full reporting of outcomes
☒	☐	Estimates of effect sizes (e.g. Cohen's *d*, Pearson's *r*), indicating how they were calculated
☐	☒	Clearly defined error bars<br>*State explicitly what error bars represent (e.g. SD, SE, CI)*

Our web collection on statistics for biologists may be useful.

Software and code

Policy information about availability of computer code

Data collection	No data was excluded from the analysis.
Data analysis	All statistical analyses were performed using either Prism 5.04 (GraphPad Software, Inc.) or Excel. The softwares used for phylogenetic analysis protein function prediction, analysis of RNAseq and proteomics data are commercially available. The version of each software is stated in the method section.

For manuscripts utilizing custom algorithms or software that are central to the research but not yet described in published literature, software must be made available to editors/reviewers upon request. We strongly encourage code deposition in a community repository (e.g. GitHub). See the Nature Research guidelines for submitting code & software for further information.

Data

Policy information about availability of data

All manuscripts must include a data availability statement. This statement should provide the following information, where applicable:

- Accession codes, unique identifiers, or web links for publicly available datasets
- A list of figures that have associated raw data
- A description of any restrictions on data availability

Gene of interest were cloned from the Roseburia intestinalis L1-82, BioProjectID PRJNA30005. Accession number of the cloned genes is provided in the results

section.
The transcriptomics and MS proteomics raw data are available from the corresponding author upon request.

Field-specific reporting

Please select the best fit for your research. If you are not sure, read the appropriate sections before making your selection.

☒ Life sciences ☐ Behavioural & social sciences ☐ Ecological, evolutionary & environmental sciences

For a reference copy of the document with all sections, see nature.com/authors/policies/ReportingSummary-flat.pdf

Life sciences study design

All studies must disclose on these points even when the disclosure is negative.

Sample size	No sample-size has been predetermined in this study.
Data exclusions	No data was excluded from the data analysis.
Replication	Technical and biological replicates are indicated in the respective figure legends.
Randomization	The mice we used in the study were born on the same days and they were randomly allocated to experimental groups.
Blinding	For the in vitro and in vivo experiments, blinding was not applicable.

Reporting for specific materials, systems and methods

Materials & experimental systems

n/a	Involved in the study	
☐	☒	Unique biological materials
☐	☒	Antibodies
☒	☐	Eukaryotic cell lines
☒	☐	Palaeontology
☐	☒	Animals and other organisms
☒	☐	Human research participants

Methods

n/a	Involved in the study	
☒	☐	ChIP-seq
☒	☐	Flow cytometry
☒	☐	MRI-based neuroimaging

Unique biological materials

Policy information about availability of materials

Obtaining unique materials	Acetylated galactoglucomannan was generated in house and is available from the corresponding author on reasonable request.

Antibodies

Antibodies used	Alexa Flour 488 goat anti rat IgG (ThermoFisher) Cat# A-11006<br>A custom made polyclonal antibody was raised against the recombinant RiGH26 proteins in rat (Eurogentec)
Validation	RiGH26, polyclonal; the antibody recognizes a surface endomannanase of Roseburia intestinalis L1-82. Species reactivity: rat.

Animals and other organisms

Policy information about studies involving animals; ARRIVE guidelines recommended for reporting animal research

Laboratory animals	In the experiment shown Fig. 6e, we used 6-week germ-free male mice that were maintained in a controlled environment in plastic flexible film gnotobiotic isolators under a strict 12 h light cycle. Animals received sterilized water and high fiber chow (LabDiet 5013) ad libitum for three days and then they were inoculated by oral gavage with 0.2 mL of mixed bacterial culture in the gnotobiotic isolator. Mice were switched to varying 7-day diet regimes, including a fiber free chow diet (TD.140343), a custom version of the same diet containing 2.5% mannan and a a custom version of the same diet containing 7.5%. The mice were then euthanized at 45-days post-gavage after 4h fasting.

Wild animals	The study did not involve wild animals
Field-collected samples	Not applicable
